# Additional Effects of Manual Therapy to Exercise on Pain and Function in Individuals With Knee Osteoarthritis: A Systematic Review and Meta‐Analysis

**DOI:** 10.1002/ejp.70397

**Published:** 2026-10-16

**Authors:** Viviane R. da Silva, Rebecca B. Ramalho, Marcos de Noronha, Marcelo A. Bracht, Luiz Felipe Tavares, Luiz F. A. Selistre

**Affiliations:** ^1^ Physiotherapy Department of the Federal University of São Carlos São Carlos Brazil; ^2^ Physiotherapy Department of the La Trobe University – Bendigo Campus Bendigo Victoria Australia; ^3^ Physiotherapy Department of the Regional University of Blumenau Blumenau Brazil

**Keywords:** exercise, knee osteoarthritis, manual therapy, pain, physical therapy modalities

## Abstract

**Objective:**

This systematic review aimed to analyse the additional effects of manual therapy (MT) to exercise (EX) compared to EX alone on pain, function, and PPT in individuals with knee osteoarthritis (KOA).

**Methods:**

Six databases (MEDLINE, PEDro, EMBASE, CENTRAL, CINAHL, and Web of Science) were searched from inception up to May 2026. Two independent reviewers selected randomized clinical trials. Standardized mean differences (SMD) and 95% confidence intervals (CI) were calculated by random‐effects for meta‐analyses. The review was registered in PROSPERO (CRD42024525169), and the certainty of a body of evidence was classified using GRADE.

**Results:**

Twenty‐nine studies were included. MT combined with EX, compared to EX alone, reduced pain in the immediate (mean difference [MD] = −0.53; 95% CI = −0.97 to −0.09 very low certainty of evidence), short‐term (MD = −1.30; 95% CI = −1.74 to −0.87 very low certainty of evidence), and medium‐term (MD = −0.98; 95% CI = −1.86 to −0.10 moderate certainty of evidence). Function also improved in the short‐term (SMD = −0.89; 95% CI = −1.49 to −0.29 very low certainty of evidence) and medium‐term (SMD = −0.62; 95% CI = −1.13 to −0.10 very low certainty of evidence). However, no significant long‐term effects were observed for either pain and function. No eligible studies assessing pressure pain threshold (PPT) were identified.

**Conclusions:**

An additional effect of MT to EX was observed; however, the certainty of the evidence was very low to low. MT can be considered an adjunctive intervention in KOA, as it appears to provide short‐duration effects on both pain relief and functional outcomes, but does not seem to extend to the long term.

**Significance Statement:**

This systematic review identifies that MT can be considered a complementary intervention when combined with EX, which appears to provide immediate, short‐ and medium‐term effects, both in pain relief and functional outcomes in KOA. The use of MT with EX may assist patients at the beginning of the rehabilitation process. However, its benefits do not appear to extend to the long term, highlighting that MT can serve as a strategy to support exercise, particularly during the initial phase of treatment.

**Abbreviations**
6MWT: 6‐Minute Walk Test
CI: confidence interval
EX: exercise
GRADE: grading of recommendations, assessment, development, and evaluations
KOA: knee osteoarthritis
MCID: minimal clinically important difference
MD: mean difference
MT: manual therapy
OA: osteoarthritis
PEDro: physiotherapy evidence database
PPT: pressure pain threshold
PRISMA: preferred reporting items for systematic reviews and meta‐analyses
PROSPERO: International Prospective Register of Ongoing Systematic Review
SMD: standardized mean difference
TUG: time up and go test

## 1 Introduction

Knee osteoarthritis (KOA) is a degenerative disease that leads to reduced function, joint stiffness, and muscle weakness, negatively impacting quality of life (Cross et al. 2014; Hunter and Bierma‐Zeinstra 2019; Long et al. 2022; Litwic et al. 2013). Pain, disability, and impaired physical performance remain the main complaints in KOA (Dainese et al. 2022). Moreover, altered pain processing mechanisms, commonly referred to as pain sensitization (peripheral and central), may be present in KOA (Fingleton et al. 2015; Arant et al. 2022). Individuals with KOA exhibit a lower pain threshold, indicating heightened sensitivity; identifying this is relevant for guiding therapeutic approaches and has been investigated using pressure pain threshold (PPT) (Arant et al. 2022; Carlesso 2023; Fingleton et al. 2015).

Accordingly, the primary goals of treatments are to reduce pain and improve function (Charlesworth et al. 2019). Current clinical practice guidelines recommend interventions such as patient education, exercise (EX), and weight loss interventions (when necessary) for the treatment of KOA (Kolasinski et al. 2020; Bannuru et al. 2019; Moseng et al. 2024). Among these interventions, manual therapy (MT) is commonly used as an adjunct to manage pain and function in chronic musculoskeletal conditions (Li et al. 2022). This intervention encompasses a wide range of techniques, including joint mobilization, joint manipulation, soft tissue techniques (manual or instrument‐assisted), massage, and acupressure (IFOMPT Standards Document 2016).

However, as highlighted by Gray et al. (2022) adjunctive treatments, including MT, show substantially more variability across guidelines, reflecting differences in interpretation of the available evidence and contributing to clinical uncertainty (Gray et al. 2022). For instance, while some guidelines support the use of MT as an adjunct (Nelligan 2023), others recommend conditionally against it (Kolasinski et al. 2020) or even associated with EX, conditionally (Bannuru et al. 2019). These discrepancies create challenges for healthcare professionals seeking to integrate guideline‐informed care into clinical practice, given that MT are used and generally well‐accepted by patients. Given this lack of consensus, it is important to conduct research evaluating whether MT meaningfully enhances the effects of EX for KOA. Systematic reviews and other clinical practice guidelines remain contradictory in their recommendations regarding the use of MT in combination with EX for KOA (Lin et al. 2020; Moseng et al. 2024; Xu et al. 2017; Runge et al. 2022; Anwer et al. 2018; Tsokanos et al. 2021; French et al. 2022). These recommendations are based on low‐quality evidence and patient preference regarding the intervention (Nelligan 2023; Kolasinski et al. 2020; Bannuru et al. 2019; Moseng et al. 2024).

Although previous studies have demonstrated benefits of MT in essential outcomes, such as pain, function, and functional tests related to daily activities, systematic reviews have reported inconsistent results, mainly due to the methodological limitations (Runge et al. 2022; Anwer et al. 2018; Tsokanos et al. 2021; French et al. 2022). The 2022 Cochrane review concluded that current evidence is insufficient to determine whether MT provides additional benefits beyond exercise due to the low certainty of the evidence (French et al. 2022). Other studies suggest short‐term benefits for pain and function; however, they present limitations related to heterogeneity of techniques, lack of dosage standardization, and comparisons with different control groups. Similarly, another review including 19 studies on knee and hip osteoarthritis reported difficulties in isolating the additional benefit of MT due to these methodological limitations (Runge et al. 2022).

Since then, approximately 22 new randomized clinical trials have been published, which may meaningfully update and potentially change the existing evidence regarding the additional effects of MT when combined with EX compared with EX alone. Furthermore, the effects of adding MT to EX on nociceptive sensitization have not yet been investigated in KOA. As altered nociceptive processing, involving peripheral and central sensitization mechanisms, may contribute to pain persistence and treatment responses, evaluating this outcome may provide additional insights into the potential mechanisms underlying the effects of MT combined with EX (Bialosky et al. 2017, 2018). Thus, it is necessary to systematically synthesize the newly available evidence and provide an updated and comprehensive evaluation of the additive effects of MT combined with EX on pain, function, and PPT.

Therefore, this systematic review aimed to synthesize the evidence regarding the additional effects of MT associated with EX compared to EX alone on pain, function, and PPT in individuals with KOA, in the immediate, short‐, medium‐, and long‐term. This updated synthesis may contribute to clinical decision‐making by offering professionals a more current and robust evidence base to guide practice.

## 2 Methods

This systematic review followed the Preferred Reporting Items for Systematic Reviews and Meta‐Analyses (PRISMA 2020) (Page et al. 2021, 2022; Higgins et al. 2024). The protocol was prospectively registered in the International Prospective Register of Ongoing Systematic Reviews (PROSPERO) under number CRD42024525169.

### 2.1 Search Strategy

Searches were conducted in six databases (MEDLINE/PubMed, PEDro, EMBASE, CENTRAL, CINAHL, and Web of Science) for studies with no language restrictions and published up to May 26, 2026 (Michaleff et al. 2011) (Appendix S1).

### 2.2 Data Screening

Two independent reviewers screened the titles and abstracts retrieved from the searches. Full‐text articles were then assessed for eligibility. Discrepancies were resolved by consultation with a third reviewer experienced in systematic reviews. The Rayyan tool (www.rayyan.ai) was used to detect duplicates and manage the screening process. Reasons for full‐text exclusions were documented in detail (Appendix S2).

### 2.3 Eligibility Criteria

Studies were included if they met the following criteria: (1) randomized controlled trial design; (2) participants diagnosed with KOA or a mixed population with KOA and hip osteoarthritis (Altman et al. 1986, 1991); (3) intervention involving manual therapy techniques, based on the IFOMPT definition (Appendix S13), associated with EX (Anwer et al. 2018); (4) comparison group receiving the identical EX as the intervention group, without MT; (5) assessment of pain intensity (by numerical scales), self‐reported function (by WOMAC—Western Ontario and McMaster Universities Osteoarthritis Index) or PPT; (6) Outcome assessments conducted immediately after the intervention; within 4 weeks (short‐term); between 4 weeks and 6 months (medium‐term); and after 6 months (long‐term).

Studies were excluded if participants had other rheumatology conditions or other joint conditions (e.g., rheumatoid arthritis); participants who had undergone knee or hip surgery; we also excluded when the study was available as abstract, protocols, conference proceedings, posters, or unpublished data.

### 2.4 Quality Assessment

The methodological quality of included studies was assessed using the PEDro scale. For studies not already indexed in the PEDro database, two independent reviewers performed the assessment. The PEDro scale has been validated for Brazilian Portuguese, and is a reliable instrument to classify the methodological quality of studies (Albanese et al. 2020; Shiwa et al. 2011; Maher et al. 2003). They were classified with low quality methodology (< 4), acceptable quality (4–5), good quality (6–8), and excellent quality (9–10) (Yamato et al. 2017). Each item is scored as “yes” when the criteria established by the checklist are clearly met and “no” when the criteria are not met or when the study does not provide sufficient information for a definitive judgement. Disagreements were resolved through discussion with a third reviewer.

### 2.5 Data Extraction

Two independent reviewers (VRS and RBR) extracted data using a standardized excel spreadsheet. We extracted the following data: study identification (e.g., author, year), sample size, participant characteristics, clinical condition, intervention and comparison group descriptions, outcome measures, intervention duration, frequency, group means, standard deviations, and change scores when available. In cases of missing or inadequately reported data, we attempted to contact the corresponding authors to obtain additional information.

### 2.6 Data Analysis

Meta‐analyses were conducted for the primary outcomes (pain, function, and PPT) and the secondary outcomes (performance‐based tests and proprioception) at the specified time points: immediate effects (reassessment immediately after intervention), short‐term effects (reassessment up to 4 weeks after intervention), medium‐term effects (reassessment 4 weeks–6 months after intervention), and long‐term effects (reassessment more than 6 months after intervention). The software Review Manager (RevMan—version 5.4.1) was used for all analyses.

Mean differences (MD) were calculated for outcomes measured with the same instruments across studies, and standardized mean differences (SMD) were calculated when different instruments or units were applied. For both, 95% Confidence Interval (CI) was also calculated. For continuous outcomes that presented MD, the effect size was classified as small (MD < 10% of the scale), medium (MD 10%–20% of the scale) or large (MD > 20% of the scale) (Cohen 1992). For continuous outcomes that presented SMD (Hedges' *g*) the effect size was small (SMD = 0.2), medium (SMD = 0.5) and large (SMD = 0.8) (Higgins et al. 2024). We evaluated the clinical relevance of the MD using the minimum clinically important difference (MCID) (Mostafaee et al. 2022, 2023). The cut‐offs for the functional test result are Timed Up and Go Test (TUG) (2.82 s), 30s Chair Sit‐to‐Stand (2,5 stands), 40 m Fast Paced Walk Test (40 m FPWT) (0.21 m/s), 6‐Minute Walk Test (6MWT) (61 m) and Stair Climb Test (3.21 s) (Terluin et al. 2022; Mostafaee et al. 2022, 2023).

The heterogeneity of the studies was analysed using the *χ* ^2^ test and the *I* ^2^ values. *I* ^2^ values were interpreted as no heterogeneity (close to 0%), low (0%–25%), moderate (25%–50%), and high (50%–90%).

For continuous outcomes, change score values were preferentially extracted when reported. When baseline and post‐intervention values were available, change scores were calculated using the RevMan calculator according to the recommended Cochrane methodology. Studies with three or four intervention groups, where groups of interest were conceptually similar, were combined in the Revman 5.4.1 software, following the recommendations of the Cochrane Handbook (Chapter 23 Section 3.4).

### 2.7 Certainty of the Evidence

The Grading of Recommendations Assessment, Development and Evaluation (GRADE) approach was used to assess the certainty of the evidence (Chapter 15). The certainty of evidence is categorized into four levels: high, moderate, low, or very low. In randomized controlled trials, certainty begins at the “high” level and may be downgraded based on five domains: risk of bias, inconsistency, indirectness, imprecision, and publication bias. The certainty of the evidence is downgraded when there was a serious or very serious risk in one or more of these domains.

1. Risk of bias: Was downgraded by 1 level if more than 25% of participants were part of studies with a high risk of bias (PEDro score < 6) and downgraded by 2 levels if more than 50% of participants were part of studies with a high risk of bias.
2. Inconsistency: Was downgraded by 1 level if heterogeneity was visually presented or if *I* ^2^ was greater than 50%. Inconsistency was downgraded by 2 levels if considered serious (due to heterogeneity in the *I* ^2^ test greater than 75%).
3. Indirectness: In the case of generalized results, it was downgraded by 1 level if more than 50% of participants were outside the target group. There is no two‐level downgrade for this item.
4. Imprecision: We used the MCID for continuous outcomes based on the GRADEpro guidance. Studies with small sample sizes, few events, or wide confidence intervals (CIs) around the effect estimate were considered imprecise.
  For continuous outcomes:
  - the certainty was downgraded by one level if the total sample size was < 400;
  - the certainty was downgraded by one level if the 95% CI excluded any effect but the effect estimate was small (SMD ≤ 0.5), or if the CI exceeded 10% of the scale in either direction.
  When both (A) and (B) were present, the evidence was downgraded by two levels.
5. Publication bias: Was downgraded by 1 level if at least 10 studies included in this review, with the same intervention comparison. We considered publication bias when studies were uniformly small and/or had significant conflicts of interest. For outcomes with more than 10 studies, a funnel plot would be generated.

The certainty of evidence was classified as high, moderate, low, or very low. In case of disagreement, a third researcher was contacted for discussions to reach a common decision.

### 2.8 Sensitivity Analysis

Depending on the degree of statistical heterogeneity, the average effect size of the meta‐analyses was estimated using random‐effects models or fixed‐effects models (fixed effects for heterogeneity levels below 50%). We visually analysed the forest plot to identify potential outliers, explored sources of heterogeneity, and performed sensitivity analyses (for primary outcomes), removing studies with a high risk of bias or those considered outliers, according to the Cochrane Handbook recommendations (Deeks et al. 2024).

## 3 Results

### 3.1 Study Selection

Initial searches retrieved 3973 records, of which 22 met the eligibility criteria and were included in the review (Figure 1). Studies included in previous reviews were also selected by the authors if they were not included in the search strategy. In this case, seven studies were identified manually from previous systematic reviews.

**FIGURE 1:**
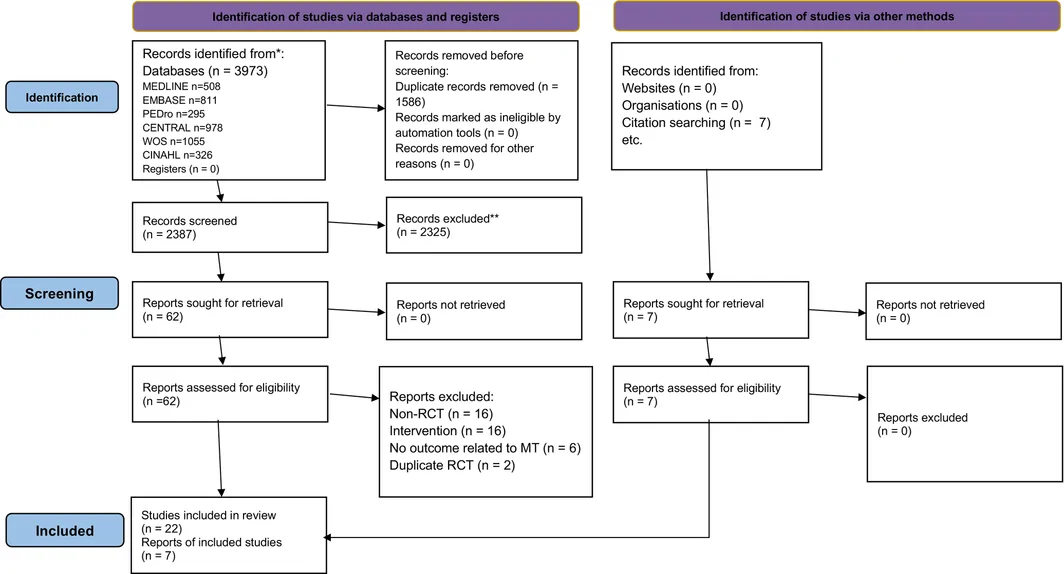
Flowchart of the study selection.

The total sample size across all included studies was 2289 participants. Two studies included mixed populations of participants with KOA (*n* = 113) and hip OA (*n* = 93) (Abbott et al. 2013, 2019). One study included only women (*n* = 45) (Lalnunpuii et al. 2017), while seven studies only reported the total number of participants (Kulkarni and Kamat 2017; Mishel and Shenouda 2013; Lalit et al. 2012; ko, et al. 2009; Godoy et al. 2014; Cheawthamai et al. 2014; Zeya et al. 2017). Of the 29 studies, 27 were RCTs and two were pilot RCTs (Dwyer et al. 2015; Rojas‐Vidal et al. 2018). Of the 29 studies included, 28 were published in English and one study was published in Spanish (Rojas‐Vidal et al. 2018). A detailed summary of all included studies is provided in Table 1.

**TABLE 1:** Characteristic of the studies.

Study identification (Country)	Sample size	Clinical condition	Groups	Intervention group descriptions	Comparison group descriptions	Outcome measures	Intervention duration	Frequency	Follow‐up
Abbott et al. 2013	206	KOA 113 and HOA 93 (W114 and M92)	Control 51 MT 54 EX 51 EX + MT 50	Aerobic Strengthening Stretching Neuromuscular control	Joint manipulation Joint mobilization	WOMAC TUG SIT AND STAND 30SEC WALK 40 METERS	50 min	7 in 9w; 2 in the 16w	1y
Abbott et al. 2015	66	KOA 66 (W46 and M29)	EX 19 EX + B 19 EX + MT 18 EX + MT + B19	Aerobic Strengthening Stretching Neuromuscular control	Joint manipulation Joint mobilization	WOMAC NPRS TUG SIT AND STAND 30SEC WALK 40 METERS	45 min	Without B: 12 em 9w; With B: 8 in 9w; 2 in the 5 m; 1 in the 8m; 1 in the 11m	1y
Abbott et al. 2019	206	KOA 113 and HOA 93 (W114 and M92)	Control 51 MT 54 EX 51 EX + MT 50	Aerobic Strengthening Stretching Neuromuscular control	Joint mobilization Joint manipulation with and without trust	WOMAC TUG SIT AND STAND 30SEC WALK 40 METERS NPRS	50 min	7 in 9w 2 in the 16w; 1 in the 54w	1y, 2y
Altınbilek 2018	85	KOA 85 (W76 and M9)	EX + MT 41 EX 44	Strengthening Stretching	Self‐mobilization of joints Self‐acupressure	WOMAC VAS WALK 50 METERS ROM	NR	2 in 2w	4w
Azlin and Lyn 2011	13	KOA 13 (W11 and M2)	EX 6 EX + MT 7	Home exercises	Joint mobilization	VAS STEP	NR	8 in 4w	4w
Cheawthamai et al. 2014	43	KOA 43 NR	EX + MT 21 EX 22	Strengthening Stretching Range of motion	Joint self‐mobilization Soft tissue manipulation	VAS ROM KOOS 6MWT	NR	NR	2w, 4w, 12w
Deyle et al. 2005	134	KOA 134 (W88 and M46)	EX + MT 66 EX 68	Strengthening Stretching	Joint mobilization Soft tissue manipulation	6MWT WOMAC ROM	NR	8 in 4w	4w, 8w, 1y
Dudonienė et al. 2025	63	KOA (W43 and M20)	EX EX + MT	Aerobic Strengthening Stretching	Joint mobilization	VAS ROM WOMAC	30‐45 min	24 in	18d
Dwyer et al. 2015	78	KOA 78 (W49 and M29)	MT 26 EX 26 EX + MT 26	Strengthening Stretching + Patient education	Joint mobilization Joint manipulation with Soft tissue manipulation	WOMAC ROM OTE	2 h	6 in 4w	5w
Fitzgerald et al. 2016	300	KOA 300 (W199 and M101)	MT + EX 75 MT + EX + B 74 EX + B 76 EX 75	Aerobic Strengthening Stretching Neuromuscular control	Joint mobilization Soft tissue manipulation	WOMAC TUG NPS SIT AND STAND 30SEC WALK 40 METERS	45 min–1 h	12 in 9w	9w, 1y
Gandotra et al. 2025	38	KOA 38 (W29 and H9)	EX 20 EX + MT 18	Conventional exercises and stretching	Instrumental soft tissue manipulation	NPRS WOMAC SIT AND STAND 30SEC ROM	NR	6 in 2w	2w
Godoy et al. 2014	18	KOA 18 NR	EX + MT 9 EX 9	Strengthening Aerobic	Massage	VAS WOMAC TUG	30–40 min	2 in 6w	4w, 12w
Guo et al. 2021	202	KOA 202 (W105 and M97)	Control 52 EX 50 MT 49 EX + MT 51	Warm‐up Aerobics Strengthening	Acupressure	WOMAC VAS ROM TUG WALK 40 METERS	NR	120 in 8w	8w, 16w
Hasan et al. 2026	50	KOA 50 (W25 and M25)	EX 25 EX + MT 25	Isometric training	Mobilization with movement	VAS WOMAC	30–40 min	30 in 6w	1w, 3w, 5w
Heggannavar and Gupta 2015	60	KOA 60 (W48 and M12)	EX conventional + MT(mobilization with movement) 20 EX proprioceptive and conventional + MT(mobilization with movement) 20 EX 20	Strengthening Balance + Short waves	Joint mobilization with movement	ROM VAS WOMAC PROPRIOCEPTION	NR	14 in 2w	1w, 2w
Iqbal et al. 2024	126	KOA 126 (W76 and H50)	EX 42 EX + MT 42 MT 42	Proprioceptive exercises + heating pad	Mulligan traction	ROM WOMAC NPRS	1 h	24 in 8w	8w
Jeyakumar et al. 2017	60	KOA 60 (W29 and M31)	EX + MT (maitland) 20 EX + MT (mobilization with movement) 20 EX 20	Strengthening + Hot compress	Joint mobilization mobilization with movement	VAS ROM	NR	NR	3w
ko et al. 2009	35	KOA 35 NR	EX + MT 18 EX 17	Stretching Resistance	Joint mobilization	ROM STRENGTH PROPRIOCEPTION WALK 10 METERS	40 min	24 in 8w	8w
Kulkarni and Kamat 2017 (USA)	30	KOA 30 NR	EX 15 EX + MT 15	Strengthening Stretching + TENS	Joint mobilization mobilization with movement	VAS 6MWT	NR	3 in 3d	3d
Lalit et al. 2012 (India)	90	KOA 90 NR	EX + MT (maitland) 30 EX + MT (mobilization with movement) 30 EX 30	Strengthening Balance	Maitland joint mobilization; Joint mobilization mobilization with movement	VAS WOMAC PROPRIOCEPTION	NR	120 in 8w	5d
Lalnunpuii et al. 2017	45	KOA 45 (W45)	EX + MT (mobilization with movement) 15 EX + MT (maitland) 15 EX 15	Strengthening Stretching	Joint mobilization mobilization with movement Joint mobilization maitland	VAS WOMAC	NR	12 in 4w	4w, 5w
Mehwish et al. 2023	66	KOA 66 (W47 and M19)	MT + EX (knee and hip) 22 EX (knee and hip) 22 EX (knee) 22	Strengthening Stretching	Joint mobilization	VAS FSST	30–40 min	8 in 4w	4w
Mishel and Shenouda 2013	45	KOA 45 NR	EX + MT (mobilization with movement) 15 EX + shock waves 15 EX 15	Strengthening Stretching	Joint mobilization mobilization with movement	VAS WOMAC ROM	NR	12 in 4w	4w
Nafees et al. 2023	48	KOA 48 (W37 and M11)	EX + MT 24 EX 24	Strengthening Stretching FNP + Cryotherapy	Joint mobilization mobilization with movement	VAS KOOS AKET	30 min	12 in 4w	4w
Nigam et al. 2021 (India)	40	KOA 40 (W25 and M15)	EX + MT 20 EX 20	Strengthening Resistance + Moist heat	Joint self‐mobilization mobilization with movement	WOMAC VAS TUG STEP NPRS	45 min	6 in 2w	2w, 12w, 6 m
Reza et al. 2021	32	KOA 32 (W13 and M19)	EX + MT 16 EX 16	Strengthening Stretching	Joint mobilization Joint manipulation	WOMAC NPRS	NR	3 in 2w	2w, 4w
Rojas‐Vidal et al. 2018	20	KOA 20 (W18 and M2)	EX + MT 10 EX 10	Aerobic Strengthening + Hot compresses	Joint mobilization	WOMAC VAS ROM 6MWT	40 min	10 in 4w	4w
Ughreja and Shukla 2017	30	KOA 30 (W22 and M8)	EX + MT(mobilization with movement) 15 EX 15	Strengthening Stretching + Short waves	Joint mobilization mobilization with movement	VAS WOMAC 6MWT SATISFACTION	NR	7 in 1w	1w
Zeya et al. 2017	60	KOA 60 NR	EX + MT 30 EX 30	Strengthening Stretching Aerobic	Joint mobilization Soft tissue manipulation	WOMAC VAS LIKERT SCALE	NR	NR	3w

Abbreviations: 6MWT, 6‐Minute Walk Test; AKET, Active Knee Extension Test; B, booster session; D, day; EX, exercise; FSST, Four Square Step Test; KOOS, knee injury and osteoarthritis outcome score; M, month; MT, manual therapy; NPRS, Numeric Pain Rating Scale; NR, not reported; OTE, overall therapy effectiveness; PNF, proprioceptive neuromuscular facilitation; TENS, transcutaneous electrical nerve stimulation; VAS, Visual Analog Scale; W, week.

Four authors were contacted by email and through ResearchGate during the data extraction period to obtain the missing data. However, we received no response from any of them; therefore, studies with missing data were not included in the analysis.

### 3.2 Description of the Interventions

The most frequently used MT techniques were: joint mobilizations, mobilization with movement, which were investigated in 25 studies. Soft tissue techniques, including soft tissue manipulation (manual and instruments), massage, and acupressure, were investigated in 10 studies, while joint manipulations (with or without thrust) were applied in five studies. Most of the MT techniques were therapist‐delivered in 22 studies (76%) and three studies (11%) were self‐administered (Cheawthamai et al. 2014; Nigam et al. 2021; Guo et al. 2022; Altınbilek 2018), and four studies (13%) did not report administered (ko et al. 2009; Zeya et al. 2017; Mehwish et al. 2023; Reza et al. 2021). The duration of the session was not reported in 16 studies (55%), but for those that did report treatment duration, session length was on average 50 min (30‐min interval—2 h). The frequency of MT sessions varied among studies, for instance one study applied daily sessions, 16 studies applied MT weekly, and seven studies also applied weekly sessions with booster interventions (Table 1 and Appendix S3).

### 3.3 Methodological Quality

The methodological quality of the included studies showed a mean score of 6. The items with the lowest scores were blinding of participants and therapists, performed in 55% of the studies. The intention‐to‐treat analysis and concealment of allocation were performed in 65% of the included studies. A detailed methodological quality assessment of each study is provided in Table 2.

**TABLE 2:** Methodological quality assessment—PEDro.

Studies (country)	1	2	3	4	5	6	7	8	9	10	11	Total score
Abbott et al. 2013	Yes	Yes	Yes	Yes	No	No	Yes	Yes	Yes	Yes	Yes	8
Abbott et al. 2015	Yes	Yes	Yes	Yes	No	No	Yes	Yes	Yes	Yes	Yes	8
Abbott et al. 2019	Yes	Yes	Yes	Yes	No	No	Yes	Yes	Yes	Yes	Yes	8
Altınbilek 2018	Yes	Yes	No	Yes	No	No	Yes	Yes	No	No	Yes	5
Azlin and Lyn 2011	Yes	Yes	No	No	No	No	Yes	No	No	Yes	Yes	4^a^
Cheawthamai et al. 2014	Yes	Yes	Yes	Yes	No	No	No	No	No	Yes	Yes	5
Deyle et al. 2005	Yes	Yes	Yes	Yes	No	No	Yes	Yes	Yes	Yes	Yes	8
Dudonienė et al. 2025	Yes	Yes	Yes	Yes	No	No	Yes	Yes	Yes	Yes	Yes	8^a^
Dwyer et al. 2015	Yes	Yes	Yes	Yes	No	No	Yes	Yes	Yes	Yes	Yes	8
Fitzgerald et al. 2016	Yes	Yes	Yes	Yes	No	No	Yes	Yes	Yes	Yes	Yes	8
Gandotra et al. 2025	Yes	Yes	No	Yes	No	No	Yes	Yes	No	Yes	Yes	6^a^
Godoy et al. 2014	Yes	Yes	Yes	Yes	No	No	Yes	Yes	Yes	Yes	Yes	8
Guo et al. 2021	Yes	Yes	No	Yes	No	No	No	Yes	No	Yes	Yes	5
Hasan et al. 2026	Yes	Yes	Yes	Yes	No	No	Yes	Yes	Yes	No	Yes	7^a^
Heggannavar and Gupta 2015	Yes	Yes	No	Yes	No	No	No	Yes	No	Yes	Yes	5^a^
Iqbal et al. 2024	Yes	Yes	Yes	Yes	No	No	Yes	Yes	Yes	Yes	Yes	8^a^
Jeyakumar et al. 2017	Yes	Yes	Yes	Yes	No	No	No	No	Yes	No	Yes	5
ko et al. 2009	Yes	Yes	No	Yes	No	No	No	Yes	No	No	Yes	4
Kulkarni and Kamat 2017	Yes	Yes	No	Yes	No	No	No	Yes	Yes	Yes	Yes	6^a^
Lalit et al. 2012	Yes	No	No	Yes	No	No	No	Yes	No	Yes	No	2^a^
Lalnunpuii et al. 2017	Yes	Yes	Yes	Yes	Yes	No	Yes	Yes	Yes	Yes	Yes	9^a^
Mehwish et al. 2023	Yes	Yes	Yes	Yes	No	No	Yes	Yes	No	Yes	Yes	7
Mishel and Shenouda 2013	Yes	Yes	No	Yes	No	No	No	Yes	Yes	Yes	Yes	6^a^
Nafees et al. 2023	Yes	Yes	Yes	Yes	No	No	Yes	Yes	No	Yes	Yes	7
Nigam et al. 2021	Yes	Yes	Yes	Yes	No	No	Yes	Yes	Yes	Yes	Yes	8
Reza et al. 2021	Yes	Yes	Yes	Yes	No	No	Yes	Yes	Yes	Yes	Yes	8^a^
Rojas‐Vidal et al. 2018	Yes	Yes	Yes	Yes	No	No	No	Yes	Yes	Yes	Yes	7^a^
Ughreja and Shukla 2017	Yes	Yes	No	Yes	No	No	No	Yes	Yes	Yes	Yes	6^a^
Zeya et al. 2017	No	Yes	No	No	No	No	No	No	No	Yes	No	2^a^
Mean												6

*Note:* Each item receives a score of “yes” if the criteria are met and “no” if they are not met or if there is insufficient information.

^a^ PEDro evaluation done manually by the authors.

### 3.4 Primary Outcome Results

The primary outcomes of pain intensity and function were analysed according to numerical scales and the WOMAC, respectively. All meta‐analyses were performed using random‐effects models, considering clinical and methodological heterogeneity across the included studies.

To ensure appropriate meta‐analysis synthesis, in Abbott et al. (2015) and Fitzgerald et al. (2016), the groups ‘EX alone’ and ‘EX alone with a booster session’, were combined, as were ‘MT + EX’ and ‘MT + EX + booster session’ (Abbott et al. 2015; Fitzgerald et al. 2016). Similarly, in Lalnunpuii et al. (2017), Jeyakumar et al. (2017) and Lalit et al. (2012), the groups “MT mobilization with movement” and ‘MT maitland’ with the same EX protocol, were combined, as well as ‘MT + EX’ compared to the ‘EX alone’ group (Lalnunpuii et al. 2017; Jeyakumar et al. 2017; Lalit et al. 2012).

Two studies were not included in the meta‐analysis for different reasons. Cheawthamai et al. (2014) provided only baseline data in tables, with follow‐up results shown in line graphs (Cheawthamai et al. 2014). Zeya et al. (2017) presented their results only using pie charts (Table 1 and Appendix S3) (Zeya et al. 2017). Corresponding authors were contacted, but without success.

#### 3.4.1 Pain Intensity

##### 3.4.1.1 Immediate

Three studies were included (Lalit et al. 2012; Godoy et al. 2014; Altınbilek 2018). The results are in favour of the additional effect of MT, with a small effect size (MD = −0.49; 95% CI = −0.92 to −0.06; *I* ^2^ = 0%) for the intensity of pain (Figure 2—Comparison A). The results showed very low certainty of evidence (downgraded due to risk of bias, imprecision, and publication bias).

**FIGURE 2:**
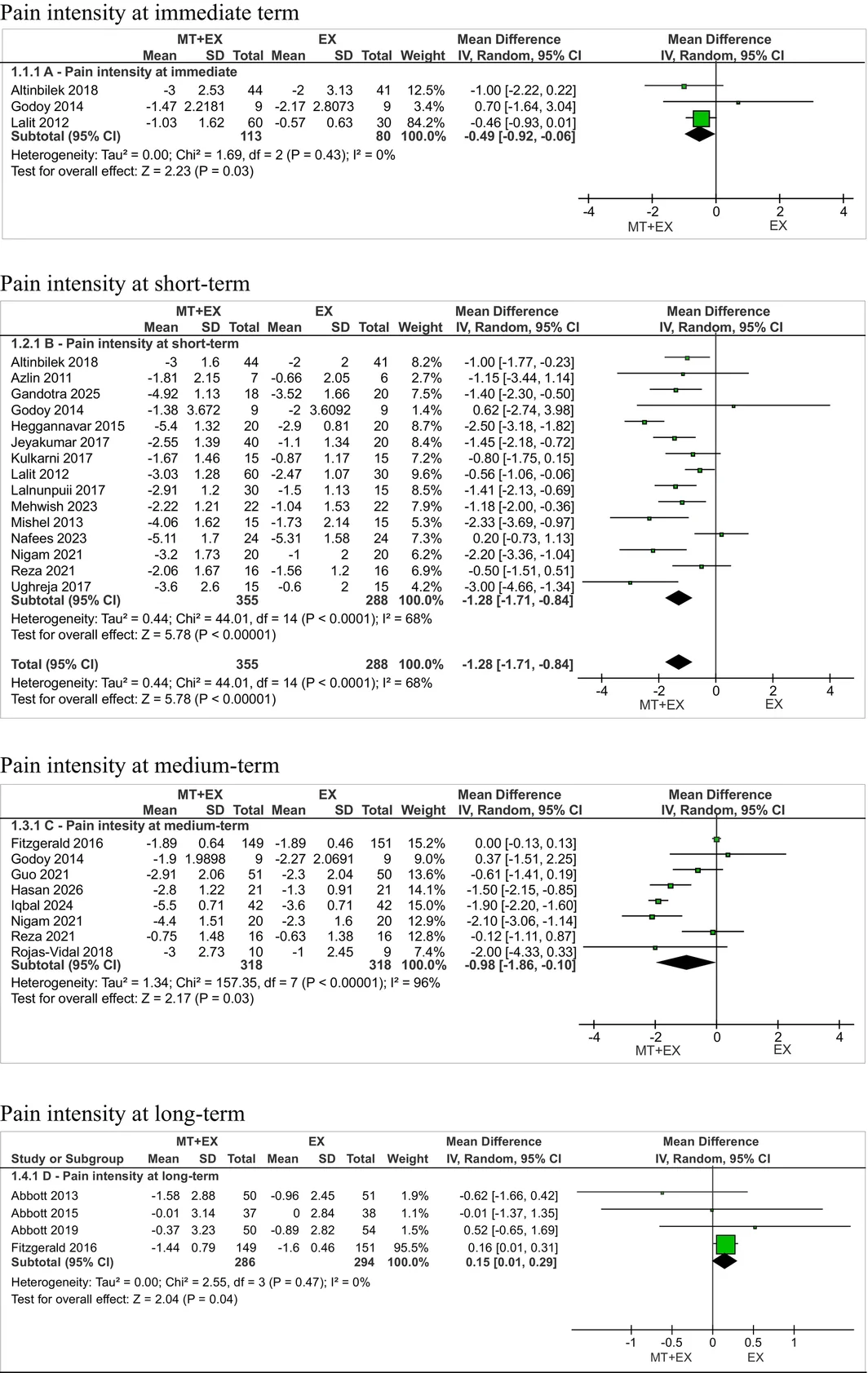
Pain intensity.

##### 3.4.1.2 Short‐Term

Fifteen studies were included (Nigam et al. 2021; Lalnunpuii et al. 2017; Kulkarni and Kamat 2017; Godoy et al. 2014; Azlin and Lyn 2011; Mehwish et al. 2023; Heggannavar and Gupta 2015; Jeyakumar et al. 2017; Nafees et al. 2023; Mishel and Shenouda 2013; Ughreja and Shukla 2017; Reza et al. 2021; Lalit et al. 2012; Altınbilek 2018; Gandotra et al. 2025). The results are in favour of the additional effect of MT, with a low effect size (MD = −1.28; 95% CI = −1.71 to −0.84; *I* ^2^ = 68%) for the intensity of pain (Figure 2—Comparison B). The results showed very low certainty of evidence (downgraded due to risk of bias and inconsistency).

The results showed high heterogeneity, so we performed a sensitivity analysis excluding one study (Godoy et al. 2014), which appeared anomalous in the funnel plot (Appendix S12). However, the results remained similar even after exclusion (MD = −1.30; CI = −1.74 to −0.87; *I* ^2^ = 70%). The results are summarized in the Appendix S11.

##### 3.4.1.3 Medium‐Term

Eight studies were included (Godoy et al. 2014; Rojas‐Vidal et al. 2018; Nigam et al. 2021; Guo et al. 2022; Fitzgerald et al. 2016; Reza et al. 2021; Iqbal et al. 2024; Hasan et al. 2026). The results are in favour of the additional effect of MT, with a small effect size (MD = −0.98; 95% CI = −1.86 to −0.10; *I* ^2^ = 96%) for the intensity of pain (Figure 2—Comparison C). The results showed low certainty of evidence (downgraded due to inconsistency and publication bias).

The results showed high heterogeneity, so we performed a sensitivity analysis excluding one study (Guo et al. 2022) which has a high risk of bias. However, the results remained similar even after exclusion (MD = −0.95; CI = −2.07 to 0.17; *I* ^2^ = 97%). The results are summarized in the Appendix S11.

##### 3.4.1.4 Long‐Term

Four studies were included (Abbott et al. 2013, 2019, 2015; Fitzgerald et al. 2016). The results are not favourable to the additional effect of MT compared to isolated EX intervention, with a small effect size (MD = 0.15; 95% CI = 0.01 to 0.29; *I* ^2^ = 0%) for the intensity of pain (Figure 2—Comparison C). The results showed moderate certainty of evidence (downgraded due to publication bias).

#### 3.4.2 Function—WOMAC

##### 3.4.2.1 Short‐Term

Eight studies were included (Lalnunpuii et al. 2017; Lalit et al. 2012; Godoy et al. 2014; Nigam et al. 2021; Deyle et al. 2005; Heggannavar and Gupta 2015; Ughreja and Shukla 2017; Reza et al. 2021). The results are in favour of the additional effect of MT, with a large effect size (SMD = −0.83; 95% CI = −1.38 to −0.29; *I* ^2^ = 83%) for the function (Figure 3—Comparison A). The results showed very low certainty of evidence (downgraded due to risk of bias, inconsistency, imprecision, and publication bias).

**FIGURE 3:**
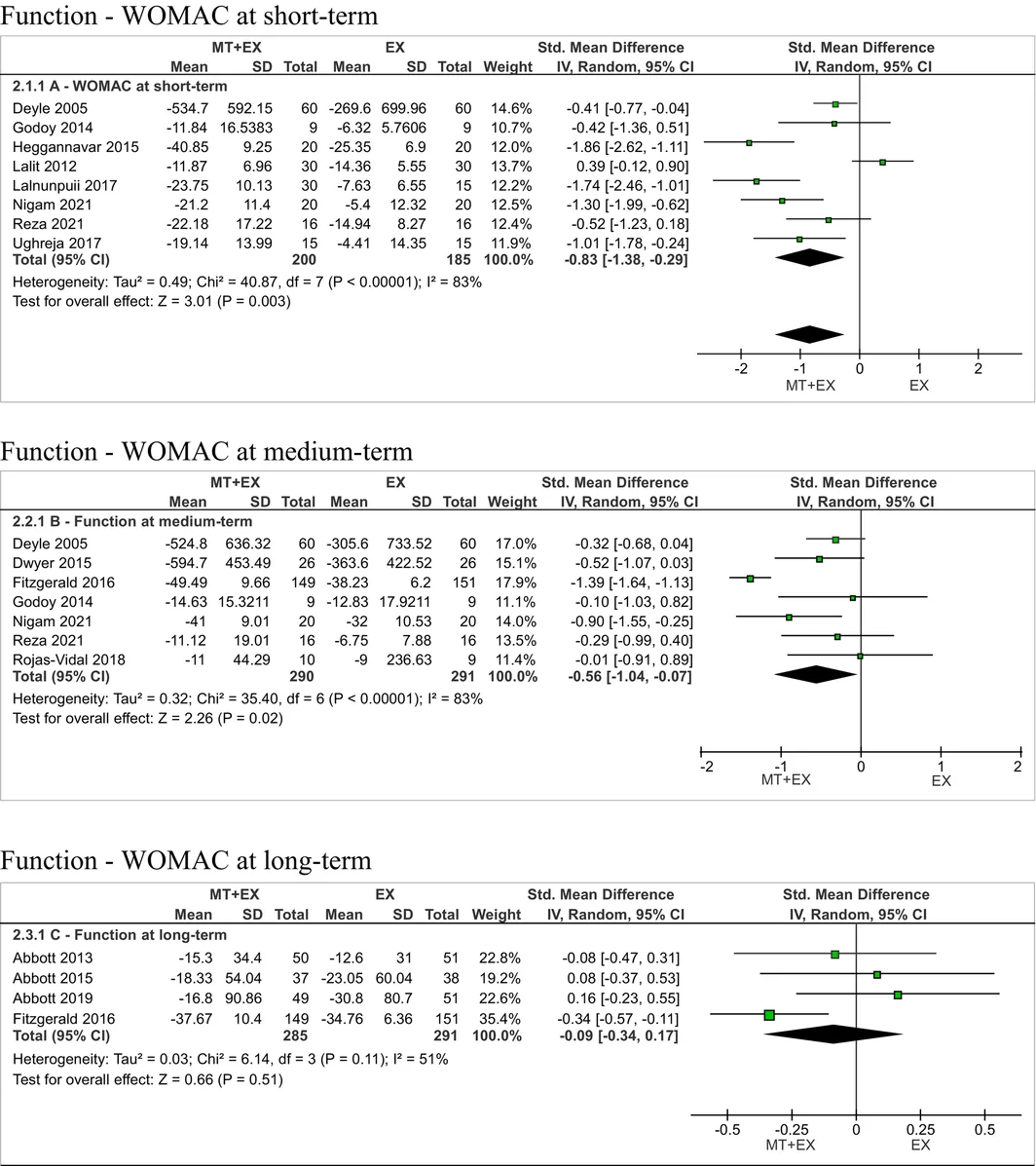
Function.

The results showed high heterogeneity, so we performed a sensitivity analysis excluding two studies (Lalit et al. 2012; Heggannavar and Gupta 2015), which has a high risk of bias. However, the results remained similar even after exclusion (MD = −0.88; CI = −1.34 to −0.43; *I* ^2^ = 65%). The results are summarized in the Appendix S11.

##### 3.4.2.2 Medium‐Term

Seven studies were included (Godoy et al. 2014; Dwyer et al. 2015; Rojas‐Vidal et al. 2018; Nigam et al. 2021; Deyle et al. 2005; Fitzgerald et al. 2016; Reza et al. 2021). The results are in favour of the additional effect of MT, with a moderate effect size (SMD = −0.56; 95% CI = −1.04 to −0.07; *I* ^2^ = 83%) for the function (Figure 3—Comparison B). The results showed very low certainty of evidence (downgraded due to inconsistency and publication bias).

##### 3.4.2.3 Long‐Term

Four studies were included (Abbott et al. 2013, 2019, 2015; Fitzgerald et al. 2016). The results indicate no difference was found between the interventions, with a small effect size (SMD = −0.09; 95% CI = −0.34 to 0.17; *I* ^2^ = 51%) for the function (Figure 3—Comparison C). The results showed very low certainty of evidence (downgraded due to inconsistency, imprecision, and publication bias).

#### 3.4.3 PPT

No study was found that investigated the effect of MT associated with EX compared to EX alone on PPT in people with KOA.

### 3.5 Secondary Outcome Results

The synthesized secondary outcomes were performance‐based tests and proprioception.

#### 3.5.1 TUG

##### 3.5.1.1 Medium‐Term

The analysis included four studies (Godoy et al. 2014; Dwyer et al. 2015; Nigam et al. 2021; Guo et al. 2022; Fitzgerald et al. 2016) and showed no difference between the interventions (MD = −1.73; 95% CI = −3.73 to 0.28; *I* ^2^ = 79%) for the TUG (Appendix S4—Comparison A). The results showed very low certainty of evidence (downgraded due to inconsistency, imprecision, and publication bias).

##### 3.5.1.2 Long‐Term

The analysis included four studies (Abbott et al. 2013, 2019, 2015; Fitzgerald et al. 2016), and the results indicate no difference between the interventions (MD = 0.27; 95% CI = −0.29 to 0.83; *I* ^2^ = 63%) for the TUG (Appendix S4—Comparison B). The results showed very low certainty of evidence (downgraded due to inconsistency, imprecision, and publication bias).

#### 3.5.2 40 m Fast Paced Walk Test

##### 3.5.2.1 Medium‐Term

The analysis included 2 studies (Guo et al. 2022; Fitzgerald et al. 2016) and showed no difference between the interventions (MD = −0.66; 95% CI = −1.81 to 0.48; *I* ^2^ = 97%) for the 40 m Fast Paced Walk Test (Appendix S5—Comparison A). The results showed very low certainty of evidence (downgraded due to risk of bias, inconsistency, and publication bias).

##### 3.5.2.2 Long‐Term

The analysis included four studies (Abbott et al. 2013, 2019, 2015; Fitzgerald et al. 2016) and showed no difference between the interventions (MD = 0.96; 95% CI = −1.27 to 3.19; *I* ^2^ = 78%) for the 40 m Fast Paced Walk Test (Appendix S5—Comparison B). The results showed very low certainty of evidence (downgraded due to inconsistency and publication bias).

#### 3.5.3 6MWT

##### 3.5.3.1 Short‐Term

The analysis included 3 studies (Kulkarni and Kamat 2017; Deyle et al. 2005; Ughreja and Shukla 2017) and showed no difference between the interventions (MD = 29.04; 95% CI = −11.89 to 69.98; *I* ^2^ = 67%) for the 6MWT (Appendix S6—Comparison A). The results showed very low certainty of evidence (downgraded due to inconsistency, imprecision, and publication bias).

##### 3.5.3.2 Medium‐Term

The analysis included 2 studies (Rojas‐Vidal et al. 2018; Deyle et al. 2005) and showed no difference between the interventions (MD = 19.22; 95% CI = −31.40 to 69.84; *I* ^2^ = 0%) for the 6MWT (Appendix S6—Comparison B). The results showed very low certainty of evidence (downgraded due to imprecision and publication bias).

#### 3.5.4 Stair Climb Test

##### 3.5.4.1 Short‐Term

The analysis included two studies (Nigam et al. 2021; Azlin and Lyn 2011) and showed no difference between the interventions (SMD = −1.22; 95% CI = −3.52 to 1.09; *I* ^2^ = 86%) for the Stair climb test (Appendix S7). The results showed very low certainty of evidence (downgraded due to inconsistency, imprecision, and publication bias).

#### 3.5.5 Proprioception

##### 3.5.5.1 Short‐Term

The analysis included two studies (Lalit et al. 2012; Heggannavar and Gupta 2015) and showed no difference between the interventions (SMD = −0.28; 95% CI = −0.64 to 0.08; *I* ^2^ = 64%) for the proprioception (Appendix S8). The results showed very low certainty of evidence (downgraded due to risk of bias, inconsistency, imprecision, and publication bias).

#### 3.5.6 30 s Chair Sit‐to‐Stand

##### 3.5.6.1 Long‐Term

The analysis included four studies (Abbott et al. 2013, 2019, 2015; Fitzgerald et al. 2016) and showed no difference between the interventions (MD = −0.10; 95% CI = −0.23 to 0.04; *I* ^2^ = 0%) for the 30 s Chair Sit‐to‐stand (Appendix S9). The results showed low certainty of evidence (downgraded due to imprecision and publication bias).

### 3.6 Publication Bias

The publication bias was assessed for all the outcomes in this systematic review following GRADE criteria. However, only one outcome (short‐term pain intensity) included ≥ 10 studies; for this reason a funnel plot was generated and visually inspected only for this outcome (Appendix S12). The plot did not show a clear pattern of asymmetry, as most studies were distributed relatively symmetrically around the pooled effect estimate, with the expected increase in dispersion among studies with higher standard errors. One study presented a large standard error and appeared more distant from the others, but its direction did not indicate any systematic trend that could bias the overall distribution. Thus, the visual inspection suggests a low likelihood of publication bias, although we acknowledge that this assessment remains limited by the inherent subjectivity of funnel plot interpretation.

## 4 Discussion and Conclusions

The objective of this study was to synthesize the additive effect of MT associated with EX when compared to EX alone in people with KOA on pain intensity, function, and PPT. The 29 included studies demonstrated the superiority of MT combined with EX in comparison to EX alone for pain intensity in the immediate, short‐term, and medium‐term. Regarding function, the combined intervention was superior to EX alone in the short‐ and medium‐terms. No other comparisons showed significant differences between groups, except for long‐term pain intensity, indicating no additional effect of MT combined with EX. Importantly, this review included 12 additional studies (Lalit et al. 2012; Cheawthamai et al. 2014; Zeya et al. 2017; Rojas‐Vidal et al. 2018; Mehwish et al. 2023; Guo et al. 2022; Jeyakumar et al. 2017; Nafees et al. 2023; Iqbal et al. 2024; Gandotra et al. 2025; Dudonienė et al. 2025; Hasan et al. 2026) in comparison to the last systematic reviews (Runge et al. 2022; Anwer et al. 2018; Tsokanos et al. 2021). Our findings reinforce those of previous systematic reviews regarding the short‐term effects on pain (Runge et al. 2022, Anwer et al. 2018, Tsokanos et al. 2021), however, this contradicts the findings of a 2022 review on the topic, which found that the addition of MT to EX in the short‐term showed no important differences in pain and function outcomes compared to EX in individuals with knee or hip osteoarthritis (French et al. 2022). In the medium‐term, eight studies were included, improving the evidence regarding the additional effect of MT to EX in pain intensity. However, the results need to be interpreted with caution, as they presented very low to moderate certainty of evidence.

We also evaluated the immediate effects of the interventions, an outcome not typically addressed in previous reviews. Although this effect was observed in only two studies, only one of which had high methodological quality, it suggests that MT may have a rapid impact on pain, though more high‐quality studies are needed to confirm these findings.

In the short‐term, the findings indicate that the addition of MT to EX promoted benefits in reducing pain intensity and improving function in individuals with KOA. These effects may be related to the neurophysiological mechanisms of MT, as well as the perception of pain relief, which favours the initial involvement of patients in the therapeutic program (Feng et al. 2023; Ferreira et al. 2024). Therefore, although MT plus EX may enhance the initial effects on pain, their contribution to improving performance‐based tests remains uncertain in the short‐term.

In the medium‐term, a significant improvement in pain and function was observed in the group that received the combined intervention, suggesting that these outcomes are sensitive even to small clinical changes. On the other hand, although performance‐based tests did not show statistically significant differences between groups, the results favour manual therapy combined with exercise. The lack of significance may be related to the limited number of studies and the small sample sizes evaluating these outcomes in the medium‐term. Additionally, the inclusion of new studies in this review changed previous conclusions regarding medium‐term pain outcomes, as earlier reviews had reported limited and inconclusive findings for this outcome at that time point (Runge et al. 2022).

For long‐term outcomes, the evidence remains limited. Only four studies were included, with three originating from the same research group. The influence of a single large study (Fitzgerald et al. 2016), which favoured EX alone, had substantial weight in the analysis. Without this study, no significant differences would have been found between the interventions. This highlights the need for additional, independent long‐term trials to clarify the effects of MT in combination with EX over extended periods.

Regarding PPT, it was not possible to conduct the analysis, as the studies addressing this outcome did not meet the inclusion criteria. Although PPT is a reliable and objective clinical measure for assessing pain sensitivity in knee osteoarthritis (KOA)—and demonstrates good discriminative ability between healthy individuals and KOA patients—its clinical utility remains uncertain, and further research is needed to establish its relevance to clinical practice (Srimurugan Pratheep et al. 2018; Suokas et al. 2012; Carlesso 2023). However, understanding the effects of MT on chronic pain sensitivity is relevant, especially including central and peripheral sensitization in individuals with KOA (Petersen et al. 2023). Studies have reported reductions in resting and walking PPT after massage and Maitland technique, respectively, compared to control groups (Pozsgai, Péter, et al. 2022; Pozsgai, Udvarácz, et al. 2022). However, these studies compared MT to placebo or no treatment, rather than to EX, which remains the standard recommendation in current guidelines (Kolasinski et al. 2020; Bannuru et al. 2019; French et al. 2022). Therefore, MT should be considered as an adjunctive therapy.

In intervention studies with EX, it is not possible to implement a placebo or blinded patients and therapists. This justifies the risk of bias in some of the included studies and the downgrading of the level of evidence in certain comparisons. Despite the low quality of the evidence, the included studies demonstrated an additional effect of MT combined with EX on pain intensity and function in individuals with KOA, confirming the findings of three similar systematic reviews (Runge et al. 2022; Anwer et al. 2018; Tsokanos et al. 2021). In addition to the lack of blinding, which led to the downgrading of the quality of the evidence, some studies did not use concealed allocation of participants and/or did not perform an intention‐to‐treat analysis.

It was not possible to perform subgroup analyses by specific MT technique, frequency, or other parameters due to the heterogeneity of techniques used and their combination among studies. Thus, it was not possible to indicate the best type of MT. Many studies combined multiple techniques, and protocols varied widely. This variation may be explained by the variety of techniques used in the studies included in this analysis. The lack of protocol standardization complicates the conduct of the trials; this means the intervention was administered in various ways—for instance, the studies by Altınbilek (2018), Cheawthamai et al. (2014), and Guo et al. (2022) utilized self‐administered joint mobilization; in another 22 studies, the intervention was performed by a therapist; whereas the studies by ko et al. (2009), Mehwish et al. (2023), Rojas‐Vidal et al. (2018), and Zeya et al. (2017) did not report who administered the MT intervention. However, five studies using joint mobilization with movement (MWM) reported positive results regarding pain intensity, WOMAC score, 6MWT, and proprioception.

Considering the primary outcomes, for pain intensity, and based on the MCID values for patients with chronic pain, only the short‐ and medium‐term effects of manual therapy exceeded the threshold for clinical relevance (0.89–1.48) (Grönkvist et al. 2024). Immediate and long‐term effects, although occasionally statistically significant, did not reach clinically meaningful levels. For physical function assessed with the WOMAC, the use of standardized mean differences precluded direct comparison with established MCID values.

Regarding secondary outcomes, when considering clinical relevance, the effect sizes observed for performance‐based outcomes generally did not exceed the MCID values (Ramalho et al. 2025). The only exception was the 40 m FPWT, whose performance proved to be clinically significant. This result converges with the findings of Ramalho et al. (2024), who demonstrated the validity and responsiveness of the 40 m FPWT, reinforcing its usefulness in detecting changes over time (Ramalho et al. 2024).

This review was conducted with methodological rigour, applying strict inclusion criteria and ensuring blinded decisions between reviewers to avoid bias. Comprehensive searches, consultation of prior reviews, and experienced researchers enhanced the scope and credibility of the findings. A substantial number of studies were included, allowing assessment of multiple clinically relevant outcomes, including functional performance tests widely used in clinical practice. However, there were limitations, such as the generalizability of our findings is limited to populations with KOA, despite the inclusion of only two studies addressing both KOA and hip osteoarthritis—though only regarding long‐term outcomes for pain, function, the 30‐s Chair Sit‐to‐Stand test, the 40‐m Fast‐Paced Walk Test, and the TUG test. Although these studies were eligible and followed methodological precedents from relevant reviews, the inability to isolate KOA‐specific data introduces some uncertainty. Second, because many trials used more than one MT technique, or combined MT with additional co‐interventions, it was not possible to perform comparative analyses of specific manual therapy techniques or to determine the optimal dose, frequency, or parameters of application. This heterogeneity limits conclusions about which components of MT may be most beneficial. Third, we included studies in which a third intervention (e.g., heat therapy) was added alongside MT and EX, provided that the comparator group received an equivalent exercise protocol. Although consistent with prior systematic reviews, this may introduce variability that could influence effect estimates. Finally, inconsistent reporting across studies (e.g., insufficient detail of intervention protocols, incomplete data, and different follow‐up periods) reduced the precision of some pooled analyses and contributed to downgrading the certainty of the evidence.

Future studies should standardize and provide comprehensive descriptions of the techniques employed to increase reproducibility from the TIDiER checklist, and incorporate new studies into subsequent reviews on the complementary effects of MT combined with EX in patients with KOA. It is also suggested to analyse the results based on the duration of the intervention and ensure that they are aligned with the timing of reassessment to improve recommendations on the duration of effect for clinicians. This combined approach may be considered an adjunctive strategy for patients with functional limitations, in whom pain modulation could potentially facilitate the performance of exercises. Additionally, future research should assess function outcomes immediately following the intervention, as well as pain processing outcomes through quantitative sensory testing to confirm whether there are benefits related to nociceptive sensitivity in this population.

This systematic review found that an additional effect of MT to EX was observed in KOA; however, the certainty of the evidence ranged from very low to moderate and should therefore be interpreted with caution. MT can be considered a complementary intervention for patients with KOA, especially when combined with EX which seems to provide immediate, short and medium‐term effects in both pain relief and functional outcomes. Using MT with EX may help patients early in their rehabilitation process. However, these effects do not seem to extend into the long term, highlighting that MT could serve as a supportive strategy to exercise, particularly during the initial management phase.

## Author Contributions

This study was designed by L.F.A.S., V.R.S., and R.B.R. The experiments were performed by V.R.S. and R.B.R. The data were analysed by V.R.S. and R.B.R., and the results were critically examined by all authors. V.R.S. had a primary role in preparing the manuscript, which was edited by R.B.R., L.F.A.S., L.F.T., M.N. and M.A.B. All authors have approved the final version of the manuscript and agree to be accountable for all aspects of the work.

## Funding

This study was funded by the Coordination for the Improvement of Higher Education Personnel (CAPES) of the State of São Paulo.

## Conflicts of Interest

The authors declare no conflicts of interest.

## Supporting information


**Appendix S1:** Search strategy.
**Appendix S2:** Reasons for full‐text exclusions.
**Appendix S3:** Details of the intervention in the included studies.
**Appendix S4:** TUG—A: medium‐term and B: long‐term.
**Appendix S5:** 40m Fast Paced Walk Test—A: medium‐term and B: long‐term.
**Appendix S6:** 6MWT—A: short‐term and B: medium‐term.
**Appendix S7:** Stair climb test—short‐term.
**Appendix S8:** Proprioception—short‐term.
**Appendix S9:** 30s chair sit‐to‐stand—long‐term.
**Appendix S10:** GRADEpro.
**Appendix S11:** Sensitivity analysis.
**Appendix S12:** Funnel plot.
**Appendix S13:** Definition of manual therapy techniques.
