# Supplementary material for: Additional Effects of Manual Therapy to Exercise on Pain and Function in Individuals With Knee Osteoarthritis: A Systematic Review and Meta‐Analysis
Source: Eur J Pain. 2026 Oct 16;30(9):e70397. doi: 10.1002/ejp.70397 (PMC13673287; doi:10.1002/ejp.70397)
Supplement: Supplementary file 1 [file EJP-30-0-s001.docx]

**Appendix S1 - Search strategy**

| # | Search PUBMED | Results  26/05/2026 |
| --- | --- | --- |
|  | POPULATION |  |
| 1 | Osteoarthritis [MeSH Terms] | 89441 |
| 2 | Osteoarthr* [Title/Abstract] | 119832 |
| 3 | Degenerative arthritis [Title/Abstract] | 1496 |
| 4 | Arthrosis [Title/Abstract] | 6072 |
| 5 | 1-4 OR | 146673 |
| 6 | Knee*[Title/Abstract] | 221736 |
| 7 | Knee [Title/Abstract] | 212051 |
| 8 | Knee Joint [MeSH Terms] | 79828 |
| 9 | 6-8 OR | 235642 |
| 10 | 5 AND 9 | 61305 |
|  | INTERVENTION |  |
| 11 | massage [MeSH terms] | 7546 |
| 12 | Acupressure [MeSH Terms] | 1178 |
| 13 | Musculoskeletal Manipulations [MeSH Terms] | 20307 |
| 14 | therapy, soft tissue [MeSH Terms] | 8873 |
| 15 | mobilizati*[Title/Abstract] | 67776 |
| 16 | manipulat*[Title/Abstract] | 276338 |
| 17 | manual therap* [Title/Abstract] | 4737 |
| 18 | thrust [Title/Abstract] | 5853 |
| 19 | neurodynamic [Title/Abstract] | 867 |
| 20 | ischemic compression [Title/Abstract] | 131 |
| 21 | Trigger point therapy [Title/Abstract] | 120 |
| 22 | Muscle-biased therapy [Title/Abstract] | 1 |
| 23 | Joint-biased therapy [Title/Abstract] | 3 |
| 24 | Neural-biased therapy [Title/Abstract] | 1 |
| 25 | OMT[Title/Abstract] | 2464 |
| 26 | chiroprac*[Title/Abstract] | 7639 |
| 27 | mulligan*[Title/Abstract] | 339 |
| 28 | mobilization with movement [Title/Abstract] | 170 |
| 29 | maitland*[Title/Abstract] | 223 |
| 30 | osteopath*[Title/Abstract] | 7798 |
| 31 | soft tissue [Title/Abstract] | 152501 |
| 32 | 11-31 OR | 527327 |
|  | STUDY DESIGN |  |
| 33 | Randomized controlled trial [Publication Type] | 661687 |
| 34 | Controlled clinical trial [Publication Type] | 752535 |
| 35 | Randomized [Title/Abstract] | 824194 |
| 36 | Placebo [Title/Abstract] | 277876 |
| 37 | Clinical trials as topic [MeSH Terms] | 426227 |
| 38 | Randomly [Title/Abstract] | 486524 |
| 39 | Trial [Title] | 368460 |
| 40 | 33-39 OR | 1887124 |
| 41 | Animals [MeSH Terms] NOT Humans [MeSH Terms] | 5455231 |
| 42 | 40 NOT 41 | 1750185 |
| 43 | 10 AND 32 AND 42  Filters applied: from 2025/6/23 - 2026/5/26 | 500  64 |

**Appendix S2 - Reasons for full-text exclusions**

| **Classification** | **Amount** | **Title** | **Reason** |
| --- | --- | --- | --- |
| Not-RCT | 1 | Auricular acupressure approach for the early stage of knee osteoarthritis | Letter to the editor |
|  | 2 | Can a program of manual physical therapy and supervised exercise improve the symptoms of osteoarthritis of the knee? | Summary |
|  | 3 | Exercise therapy, manual therapy, or both, for management of osteoarthritis of the hip or knee: 2-year follow-up of a randomized clinical trial | Summary |
|  | 4 | THE IMPACT OF MANUAL THERAPY ON ADHERENCE TO KNEE OSTEOARTHROSIS REHABILITATION PROGRAM | Summary |
|  | 5 | Quantitative effects of proprioceptive exercises and mulligan's MWM in subjects with osteoarthritis of knee-a randomized clinical trail | Summary |
|  | 6 | ROLE OF MANUAL THERAPY IN DECREASING DISABILITY CAUSED BY KNEE OSTEOARTHRITIS | Summary |
|  | 7 | Comparison of the effect of different physical therapy program in the rehabilitation of knee osteoarthritis patients | Summary |
|  | 8 | Do manual therapy or booster sessions in addition to exercise therapy for knee osteoarthritis provide additional benefits? a randomized clinical trial | Summary |
|  | 9 | Exercise, manual therapy, and use of booster sessions in physical therapy for knee OA: A multi-center randomized clinical trial | Summary |
|  | 10 | The MOA trial – Management of OsteoArthritis by exercise therapy and/or manual physiotherapy in addition to usual care | Protocol |
|  | 11 | MULTICENTER RANDOMIZED CONTROLLED TRIAL OF STANDARD PHYSICAL THERAPY VS. SHAM PT IN PERSONS WITH KNEE PAIN, MENISCAL TEAR, AND OSTEOARTHRITIS | Summary |
|  | 12 | Effect of Eccentric Strengthening Exercise in patients with Knee Osteoarthritis : a Randomized Controlled Trial | RCT registration |
|  | 13 | Comparing the Effectiveness of Aqua Walking and Mobilization Exercises on Pain, Function, and Inflammation in Osteoarthritis: a Randomized Controlled Trial | RCT registration |
|  | 14 | COMPARISON OF THE EFFECT OF DIFFERENT PHYSICAL THERAPY PROGRAM IN THE REHABILITATION OF KNEE OSTEOARTHRITIS PATIENTS | Summary |
|  | 15 | Impact of Mulligan Squeeze Technique on Meniscal Derangement in Knee Osteoarthritis Patients | RCT registration |
|  | 16 | MULTICENTER RANDOMIZED CONTROLLED TRIAL OF STANDARD PHYSICAL THERAPY VS. SHAM PT IN PERSONS WITH KNEE PAIN | Summary |
| Distinct groups | 1 | Clinic-Based Patellar Mobilization Therapy for Knee Osteoarthritis: A Randomized Clinical Trial | Control group: waiting list |
|  | 2 | A comparison of two manual physical therapy approaches and electrotherapy modalities for patients with knee osteoarthritis: A randomized three arm clinical trial | All groups with exercise + adjunctive therapy |
|  | 3 | Spa therapy in the treatment of knee osteoarthritis: a large randomised multicentre trial | Manual therapy is the smallest part of the treatment, many adjunct therapies. |
|  | 4 | Effectiveness of Maitland's Mobilization and Therapeutic Exercises with Ultrasound in Patients with Osteoarthritis of Knee | Manual Therapy Group + ultrasound and no exercise |
|  | 5 | Effects of glucosamine and chondroitin sulfate supplementation in addition to resistance exercise training and manual therapy in patients with knee osteoarthritis: A randomized controlled trial | The comparison was between glucosamine and placebo, both groups received TM+exercise |
|  | 6 | Effects of non-surgical joint distraction in the treatment of severe knee osteoarthritis | TENS + ULTRASOUND + HOT COMPRESSES + EXERCISES |
|  | 7 | Biomechanical analysis in patients with knee osteoarthritis after chinese massage combined with functional exercise | Unbalanced groups |
|  | 8 | Spa therapy in the treatment of knee osteoarthritis: THERMARTHROSE a large randomised multicenter trial | Many adjunct therapies in groups |
|  | 9 | Spa therapy in the treatment of knee osteoarthritis: A large randomized trial | Many adjunct therapies in groups |
|  | 10 | Clinical observations on the effect of Tuigua manipulation combined with quadriceps exercise for the treatment of degenerative gonarthritis | Unbalanced groups |
|  | 11 | Crenobalneotherapy (spa therapy) in patients with knee and generalized osteoarthritis: a post-hoc subgroup analysis of a large multicentre randomized trial. | Many adjunct therapies in groups |
|  | 12 | Gender differences in effects of Graston technique, active release technique, and PNF stretching on hamstring flexibility in knee osteoarthritis. | All groups with exercise + manual therapy |
|  | 13 | The effects of aromatherapy massage in menopausal women with knee osteoarthritis: A randomized controlled study. | All groups with manual therapy |
|  | 14 | Self-administered acupressure for knee osteoarthritis: a randomised controlled trial (abridged secondary publication) | All groups with exercise + manual therapy |
|  | 14 | Deep Front Line Myofascial Release Versus Novel Soft Tissue Kinetic Chain Activation Technique (K-CAT) on Pain, Radiological Patellar Position and Dynamic Knee Valgus in Knee Osteoarthritis: A Randomized Clinical Trial. | All groups with exercise + manual therapy |
|  | 15 | Effectiveness of Mobilisation with Movement (MWM) along with Usual Care for Knee Osteoarthritis: A Randomised Clinical Trial | All groups with exercise + manual therapy |
|  | 16 | Effectiveness of Patellar Mobilization and Sit-To-Stand Exercises Compared to Conventional Physiotherapy on Fall Risk and Functional Mobility in Geriatric Patients with Knee Osteoarthritis | Different exercise protocol between the groups |
| Duplicate | 1 | Effect of Manual Therapies With Supervised Exercise Protocol on Pain and Functional Disability in Patients With Knee OA | Duplicate article (article protocol included) |
|  | 2 | Efficacy of Specified Manual Therapies in Combination with a Supervised Exercise Protocol for Managing Pain Intensity and Functional Disability in Patients with Knee Osteoarthritis | Duplicate article |
| Other outcomes | 1 | Long-term cost-effectiveness of exercise therapy and/or manual therapy for hip or knee osteoarthritis: randomized controlled trial and computer simulation modelling | Socioeconomic assessment |
|  | 2 | Are manual therapy or booster sessions worthwhile in addition to exercise therapy for knee osteoarthritis: economic evaluation and 2-year follow-up of a randomized controlled trial | Socioeconomic assessment |
|  | 3 | Manual therapy, exercise therapy, or both, in addition to usual care, for osteoarthritis of the hip or knee. 2: economic evaluation alongside a randomized controlled trial | Socioeconomic assessment |
|  | 4 | Exercise therapy, manual therapy, or both, for management of osteoarthritis of the hip or knee: economic evaluation alongside a randomized clinical trial | Socioeconomic assessment |
|  | 5 | Effect of Graston technique on hamstring flexibility in knee osteoarthritis patients: A randomized controlled study. | has no pain or function outcome |
|  | 6 | Comparison of instrument-assisted soft tissue mobilization and proprioceptive neuromuscular stretching on hamstring flexibility in patients with knee osteoarthritis | has no pain or function outcome |

**Appendix S3: Details of the intervention in the included studies**

| **Author, year** | **Brief name** | **What - Procedures - MT** | **What - Procedures - EX** | **Who provided** | **When and how much** |
| --- | --- | --- | --- | --- | --- |
| Abbott, 2013 | Control 51  MT 54  EX 51  EX+MT 50 | Hip:  Mandatory interventions:  1. Long-axis hip distraction with thrust  2. Lateral hip distraction, non-thrust  3. Antero-posterior directed force to the proximal femur, non-thrust  4. Postero-anterior directed force to the proximal femur, non-thrust  5. Medial hip rotation, non-thrust  6. Soft tissue manipulation to hip and thigh musculature and fascia  7. Manual stretches to connective tissue of hip and thigh  Secondary (non-mandatory) interventions prescribed when indicated by assessment findings:  1. Knee flexion, non-thrust  2. Proximal tibio-fibular joint manipulation, thrust or non-thrust  3. Knee extension, non-thrust  4. Patellar gliding force, non-thrust  5. Ankle and talo-calcaneal joint distraction, thrust or non-thrust  6. Antero-posterior talo-crural ankle directed force, non-thrust  7. Anteroposterior directed force to distal fibula, tibiofibular joint, non-thrust  8. Soft tissue manipulation, ankle plantarflexor muscle group  9. Lumbopelvic rotation thrust manipulation  Home program of reinforcing activities:  1. Prescribed up to six range-of-motion activities to reinforce clinical interventions.  Knee:  Mandatory interventions:  1. Knee flexion, non-thrust  2. Anteroposterior directed force to the tibia, tibio-femoral joint, non-thrust  3. Knee extension, non-thrust  4. Postero-anterior directed force to the tibia, tibio-femoral joint, non-thrust  5. Patellar gliding force, non-thrust  6. Manual stretch to quadriceps, hamstring, triceps surae muscle groups  7. Soft tissue manipulation, quadriceps and peripatellar connective tissue, hamstring, hip adductor and triceps surae muscle groups  Secondary (non-mandatory) interventions prescribed when indicated by assessment findings:  1. Long-axis hip distraction with thrust  2. Side hip distraction, non-thrust  3. Anteroposterior directed force to proximal femur, non-thrust  4. Poster-anterior directed force to proximal femur, non-thrust  5. Medial hip rotation, non-thrust  6. Soft tissue manipulation to hip and thigh musculature and fascia  7. Manual stretches to connective tissue of hip and thigh  8. Ankle and talo-calcaneal joint distraction, thrust or non-thrust  9. Ankle talocrural anteroposterior directed force, non-thrust  10. Anteroposterior directed force to distal fibula, tibiofibular joint, non-thrust  11. Soft tissue manipulation, ankle plantarflexor muscle group  Lumbopelvic rotation thrust manipulation  Home program of reinforcing activities:  1. Prescribed up to six range of motion activities to reinforce clinical interventions. | 1. Aerobic exercise: up to 10 minutes cycle or walk  2. Strengthening: 3 sets of 10 repetitions of:  Hip: hip abduction; hip extension; hip lateral rotation; knee extension.  Knee: knee extension; hip extension; knee flexion. Resistance adjusted as appropriate.  3. Stretching: 60 seconds passive stretch of:  Hip: hip flexors; knee extensors; hip extensors; knee flexors; hip abductors and lateral rotators; ankle plantarflexors.  Knee: knee flexors; knee extensors; ankle plantarflexors;  4. Neuromuscular coordination control exercises: Hip or knee: 3 sets of 2 minutes of (choose from): standing weight-shifting exercises; standing balance on uneven surfaces; side-stepping, forward-backward and shuttle-walking drills; or stair walking  Secondary (non-mandatory) interventions, prescribed when indicated by assessment findings:  Hip: Trunk muscle strengthening; ankle plantarflexor muscle group strengthening; hip flexor strengthening.  Knee: ankle plantarflexor muscle group strengthening; hip abductor strengthening; lateral hip rotator strengthening; hip flexor and knee extensor stretching; trunk muscle strengthening.  Home exercise program:  Prescribed up to six of the above activities to reinforce clinical interventions. | Physiotherapists | 7 in 9W;  2 in 16W;  50min |
| Abbott, 2015 | EX 19  EX+B 19  EX+MT 18  EX+B+MT 19 | 1. Knee flexion: nonthrust physiologic motion  2. Anteroposterior-directed force to the tibia, tibiofemoral joint: nonthrust  3. Knee extension: nonthrust physiologic motion  4. Posteroanterior-directed force to the tibia, tibiofemoral joint: nonthrust  5. Patellar gliding force: nonthrust  6. Manual stretch to quadriceps, hamstrings, triceps surae muscles  7. Soft tissue manipulation: quadriceps and peripatellar connective tissue, hamstrings, hip adductors, and triceps  surae muscles | 1. Aerobic exercise: up to 10 minutes, cycle or walk  2. Strengthening: 3 sets of 10 repetitions of knee extension, hip extension, knee flexion. Resistance adjusted  as appropriate  3. Stretching: 60-second passive stretch of knee flexors, knee extensors, ankle plantar flexors  4. Neuromuscular coordination control exercises: 3 sets of 2 minutes of (choose from) standing weight shifting,  standing balance on uneven surfaces, sidestepping, forward/backward and shuttle walking drills, stair walking" | Physiotherapists | Without B: 12 in 9W;  With B: 8 in 9W;  2 in 5M;  1 in 8M;  1 in 11M.  45min |
| Abbott, 2019 | Control 51  MT 54  EX 51  EX+MT 50 | 1. Knee flexion, non-thrust  2. Proximal tibio-fibular joint manipulation, thrust or non-thrust  3. Knee extension, non-thrust  4. Patellar gliding force, non-thrust  5. Ankle and talo-calcaneal joint distraction, thrust or non-thrust  6. Antero-posterior talo-crural ankle directed force, non-thrust  7. Anteroposterior directed force to distal fibula, tibiofibular joint, non-thrust  8. Soft tissue manipulation, ankle plantarflexor muscle group  9. Lumbopelvic rotation thrust manipulation  Home program of reinforcing activities:  1. Prescribed up to six range-of-motion activities to reinforce clinical interventions.  Knee:  Mandatory interventions:  1. Knee flexion, non-thrust  2. Anteroposterior directed force to the tibia, tibio-femoral joint, non-thrust  3. Knee extension, non-thrust  4. Postero-anterior directed force to the tibia, tibio-femoral joint, non-thrust  5. Patellar gliding force, non-thrust  6. Manual stretch to quadriceps, hamstring, triceps surae muscle groups  7. Soft tissue manipulation, quadriceps and peripatellar connective tissue, hamstring, hip adductor and triceps surae muscle groups  Secondary (non-mandatory) interventions prescribed when indicated by assessment findings:  1. Long-axis hip distraction with thrust  2. Side hip distraction, non-thrust  3. Anteroposterior directed force to proximal femur, non-thrust  4. Poster-anterior directed force to proximal femur, non-thrust  5. Medial hip rotation, non-thrust  6. Soft tissue manipulation to hip and thigh musculature and fascia  7. Manual stretches to connective tissue of hip and thigh  8. Ankle and talo-calcaneal joint distraction, thrust or non-thrust  9. Ankle talocrural anteroposterior directed force, non-thrust  10. Anteroposterior directed force to distal fibula, tibiofibular joint, non-thrust  11. Soft tissue manipulation, ankle plantarflexor muscle group  Lumbopelvic rotation thrust manipulation  Home program of reinforcing activities:  1. Prescribed up to six range of motion activities to reinforce clinical interventions. | 1. Aerobic exercise: up to 10 minutes cycle or walk  2. Strengthening: 3 sets of 10 repetitions of:  Hip: hip abduction; hip extension; hip lateral rotation; knee extension.  Knee: knee extension; hip extension; knee flexion. Resistance adjusted as appropriate.  3. Stretching: 60 seconds passive stretch of:  Hip: hip flexors; knee extensors; hip extensors; knee flexors; hip abductors and lateral rotators; ankle plantarflexors.  Knee: knee flexors; knee extensors; ankle plantarflexors;  4. Neuromuscular coordination control exercises: Hip or knee: 3 sets of 2 minutes of (choose from): standing weight-shifting exercises; standing balance on uneven surfaces; side-stepping, forward-backward and shuttle-walking drills; or stair walking  Secondary (non-mandatory) interventions, prescribed when indicated by assessment findings:  Hip: Trunk muscle strengthening; ankle plantarflexor muscle group strengthening; hip flexor strengthening.  Knee: ankle plantarflexor muscle group strengthening; hip abductor strengthening; lateral hip rotator strengthening; hip flexor and knee extensor stretching; trunk muscle strengthening.  Home exercise program:  Prescribed up to six of the above activities to reinforce clinical interventions. | Physiotherapists | 7 in 9W;  2 in 16W;  1 in 54W.  50min |
| Altınbilek, 2018 | EX 44  EX+MT 41 | Patients in Group 1 received multidrug therapy (MDT) as 3-minute mobilization and 3-minute compression for the bilateral patellofemoral and tibiofemoral joints, respectively, with one-minute intervals, in addition to the exercise program. Following these applications, a two-minute bilateral lower extremity pumping technique was performed. All these applications were taught to the patient to be applied twice daily at home. | An exercise program including isometric quadriceps strengthening, straight leg raises, iliotibial band exercises, hamstring stretching, hip abductor and adductor muscle strengthening, and stretching exercises was applied as 10 repetitive sets of 3, two days a week, totaling four sessions, at the clinic, and the program was taught to patients for application twice a day at home. | Self-administered | 2 in 2W  NR |
| Azlin, 2011 | EX 6  EX+MT 7 | Individuals in the intervention group received passive joint mobilization in addition to conventional physiotherapy. Joint mobilization includes anteroposterior (AP) glide of the tibia on the femur and patella gliding in all directions. | The control group received conventional therapy alone. Individuals were also advised to continue treatment given they exercised daily at home. An exercise instruction sheet and adherence log for the home program was provided to facilitate self-practice of the exercise program. All subjects continued with their normal medications during the study period. Composed of lower limb stretches  muscles (gastrocnemius, soleus, and hamstrings), isometric quadriceps work, closed kinetic chain exercise (seated leg press or partial squat or step-up), and static cycling. | Physiotherapists | 8 in 4W  NR |
| Cheawthamai, 2014 | EX 22  EX+MT 21 | Impairment: Loss of patella glides  Self-manual intervention:  Mobilization (grades III to IV) of the patella in 5°–10° knee flexion  – Medial/lateral displacement  – Elevation  – Depression  Impairment: Loss of tibiofibular joint glides  Mobilization (grades III to IV) of the tibiofibular joint:  – Through range of motion (ROM)  – End-range knee extension  Soft tissue tightness  Soft tissue massage (circular superficial friction and/or deep-fiber friction):  – Suprapatellar + peripatellar regions  – Medial and lateral joint capsule  – Popliteal fossa  – Gastrocnemius muscle  – Iliopsoas muscle  – Tensor fascia  – Iliotibial band | Stretching exercise  Standing calf stretch; Supine hamstring stretch; Prone quadriceps stretch  Hold for 10 sec  Repeat 3 × 5 times  Range of motion exercise  Knee in mid-flexion to full-extension in supine position; Knee in mid-flexion to full-flexion in supine position 10 times per set (2 sets)  Strengthening exercise  Static quad sets in knee extension; Standing terminal knee extension; Seated leg presses; Partial squats (weight-lessened); Step-ups Hold for 10 sec Repeat 3 × 10 times | Self-administered | NR  NR |
| Deyle, 2005 | MT + EX 66  EX 68 | Manual therapy techniques, consisting of physiological and accessory passive movements, muscle stretching, and soft tissue mobilization, were applied by the physiotherapist responsible for the treatment primarily to the knee and adjacent structures. Similar manual treatments were also administered to the lumbar spine, hip, and ankle if these areas exhibited a limitation in active or passive movement and were considered to contribute to the overall dysfunction of the lower limbs. | This program consisted of active ROM exercises,  muscle strengthening, muscle stretching, and riding a stationary bicycle. A physical therapist or physical therapy technician supervised these exercises. The number of strengthening exercise bouts and stationary bicycle riding time were increased or decreased by the treating physical therapist based on subject response. | Physiotherapist | 8 NR  NR |
| Dudonienè, 2025 | MT + EX 21  EX 21 | Patients in the MT group received the same exercise program as those in the EX-only group, with the additional inclusion of knee joint mobilization. Therapeutic exercises were performed first, followed by mobilization. Joint mobilization was conducted passively and included posterior and anterior tibial glides and patellar glides in all directions, applied at a rate of two to three oscillations (grade I or II) per second for 1–2 min.  Anterior and Posterior tibial glide technique; Passive patellofemoral joint mobilization: the inferior glide technique; the lateral–medial glide technique; the superior glide technique; the medial–lateral glide technique. Movements in each direction were repeated three to six times. All glides were performed with the patient in a supine position. Patients in this group received 24 sessions (16 EX and 8 JM). | The program consisted of aerobic, strengthening, and stretching exercises. Participants cycled on a stationary bicycle for up to 10 min at a self-selected pace. The strengthening component included both isometric and isotonic exercises targeting the quadriceps, hip flexors, extensors, adductors, and abductors. Isometric exercises involved maximal voluntary contractions held for 10 s, with 10 repetitions per muscle group. Isotonic exercises were performed through a full ROM against resistance, also with 10 repetitions per exercise. Static stretching exercises focused on the gastrocnemius-soleus complex and hamstring muscles, with each stretch held for 30 s and repeated three times per muscle group. Each session lasted approximately 30 min and was performed twice daily under supervision or with guided instruction. | Physiotherapists | 32 in 18D  30min  24 in 18D  30+15min |
| Dwyer, 2015 | MT 26  EX 26  EX+MT 26 | The treatment comprised joint mobilization (grades 1-4) and joint manipulation (grade 5; high-velocity, low-amplitude thrust manipulation) of the affected kinematic chain (knee, hip, foot, and spine). | The rehabilitation program included patient education, exercise prescription and passive stretching (only on treatment days) up to the knee and other parts throughout the kinetic chain, when necessary, based on functional assessment. | Chiropractors | 6 in 4W  2h |
| Fitzgerald, 2016 | EX 75  EX+B 76  MT+EX 75  MT+EX+B 74 | During manual techniques, the joint should be pushed to its limit, employing the maximum appropriate mobilization. The therapist should achieve at least one set of grades III, IV, or V in each session. (Caution with end-of-reach (EOR) techniques for knee flexion, as a degenerative meniscus is easily aggravated in this position.) | The program begins with a 10-minute aerobic warm-up (treadmill walking or stationary bike). After the warm-up, participants performed a series of strengthening, stretching, and neuromuscular control activities (agility and balance training techniques), considered core exercises of the program. In addition to the core exercises, therapists had the option to select additional exercise activities based on the results of the initial examination. These exercises addressed strength or flexibility in the hip and ankle if deficiencies were identified in the initial examination. | Physiotherapists | 12 in 9W  45min-1h |
| Gandotra, 2025 | EX 20 EX+MT 18 | The IASTM release technique is a form of manual therapy used to diagnose and treat soft tissue injuries and restrictions. It involves the use of specialised stainless steel instruments that are gently stroked over the skin to detect and address areas of fibrous tissue, scar tissue,  or adhesions. By applying controlled pressure, the technique helps break down these abnormal tissue formations, promoting healing, increasing mobility, and reducing pain. | Straight leg raises 10 seconds×5 reps  Vastus medialis obliquus strengthening 5 seconds×10 reps  Static hip adductor exercise 5 seconds×10 reps  Hip abductor strengthening 5 seconds×10 reps  Dynamic quadriceps exercise 5 seconds×10 reps  Hamstring stretching 10 seconds×5 reps | Physiotherapist | 6 in 2W |
| Godoy, 2014 | EX+MT 9  EX 9 | The massage-therapy strokes used in Group A were pressure, muscle stripping (performed with the tip of the thumbs, and kneading. Massage-therapy strokes were applied to the different heads of the quadriceps (over 5 minutes) and to the hamstring muscles (over 5 additional minutes). | Stretching, range of motion, and muscle-strengthening exercises:  ∗ Exercise 1: increasing flexibility by knee joint  capsule-stretching exercise; patient in a sitting,  non-weight bearing position (Fig. 2)  The patient sat on the edge of the stretcher, with the knees bent at a right angle off the edge of the stretcher; the feet did not touch the ground, with a 1-kilo ankle weight on the affected leg; the patient stayed in this position for 5 minutes.  ∗ Exercise 2: Knee joint last degree extension exercise in the supine position.  With the patient in the supine position, a wedge was placed under the affected knee so it was  higher than the hip; the patient had a 30 degree bend in the knee, and tried not to rotate the leg with the toes pointing upwards; the patient gently pushed the wedge with the leg until the knee  was fully extended (starting at a 30 degree bend  in the knee) by tightening the quadriceps; the  patient completed 10 repetitions, holding the final position for 5 seconds each.  ∗ Exercise 3: Extension raising exercise  In the supine position, the patient raised the affected leg to a completely straight position (40 degree bend in the hip), held the position for 5 seconds, then slowly lowered the leg; the patient completed 10 repetitions in each session.  Exercises 2 and 3 are shown.  – Aerobic exercises: 5-minute sitting pedal exercise and 10-minute walking | Physiotherapist | 2 in 6W  30-40min |
| Guo, 2021 | Control 52  EX 50  MT 49  EX+MT 51 | The exercises from the previous group and a series of acupressure points on their own. These points included SP 9 and 10 (Yinlingquan and Xuehai), ST 34, 35 and 36 (Liangqiu, Dubi and Zusanli), EX-LE 2 and 4 (Heding and Neixiyan) and GB 34 (Yanglingquan), and their specific location. Patients were asked to massage these acupuncture points one by one, each for 5 minutes.  ST34 On a line drawn between the outside  border of the kneecap and the pelvis  bone, 2 cun above the kneecap.  ST35 On the knee immediately below the  kneecap and outside the tendon below the kneecap.  ST36 Below the knee, 3 cun below ST35, one  finger breadth outside to the front part of the lower leg bone.  EX-LE2 In the middle just above the kneecap.  EX-LE4 On the knee immediately below the  kneecap, and inside the tendon below the kneecap.  GB34 Below the outside aspect of the knee, 1  cun below the head of the fibula.  SP9 On the inside of the lower leg just below  the knee joint line.  SP10 2 cun above the kneecap on the inside muscle. | Warm-up exercises, aerobic exercises for the legs, muscle strengthening, and nerve response. | Self-administered | 120 in 8W  NR |
| Hasan, 2026 | MT + EX 25  EX 25 | The glide was applied with moderate force to achieve pain-free movement and maintained throughout the active range. Intensity was adjusted individually to ensure complete symptom relief during movement, in accordance with Mulligan principles.  *Medial glide:* Non-weight-bearing: Participant supine, while the therapist applied a medial tibiofemoral glide during active knee flexion and extension.  *Weight-bearing*: Participant stood with the affected foot on a stool while the therapist maintained the medial glide during active knee flexion–extension.  *Medial rotation:* The therapist applied a medial rotational force to the proximal tibia while the participant actively flexed and extended the knee. | Isometric strength training: Following MT or as a stand-alone intervention, participants performed isometric strengthening exercises targeting the quadriceps, hamstrings, and gluteal muscles. Participants sat with their knee flexed to 60 degrees on the Isomove device and contracted their quadriceps isometrically with maximum force for 5 s, resting 10–15 s between repetitions and 1–2 min between sets. They performed three sets of 10 repetitions, five days a week, for six weeks. Each session lasted 20–25 min. | Physiotherapists | 30 in 6W  25+15min |
| Heggannavar, 2015 | EX 20  mobilization with movement+ EX conventional 20  mobilization with movement+ EX proprioceptive, conventional 20 | Mulligans  Medial or lateral rotation MWM was performed with 3 sets of 10 repetitions and 1 session per day for 14 days. | One-legged balance, b) Blind-legged balance, c) Toe walking, d) Heel walking, e) Cross-leg swing.  In the conventional group, patients received shortwave diathermy and static exercises for the hamstrings and quadriceps. | Physiotherapists | 14 in 2W  NR |
| Iqbal, 2024 | EX 42  EX+MT 42  MT 42 | Group B, receiving MT-SLR stretch, performed  the intervention three times a week for 30 minutes per session. Each session involved three sets of 10 repetitions of straight leg lifts while applying manual traction, with a 30-second break between sets. The traction force was tailored to each participant's comfort level, with repetitions held for 15-20 seconds,  followed by a 30-second rest between sets. | The proprioceptive activities assigned to Group A included half squats, standing on one foot, walking on heels and toes (both with eyes open and closed), one-legged balance exercises with a heating pad, and knee flexion and extension exercises performed while seated. Group A participants completed these exercises three times a week for eight weeks, totaling 24 sessions. Each session lasted 45 minutes. The exercises began with low-to-moderate intensity balance and stability drills, such as single-leg stances and balance exercises on unstable surfaces, progressing from static to dynamic exercises. During one-legged balancing exercises, participants used a heating pad on the affected knee for 10-15 minutes to promote relaxation and improve blood circulation to the joint before starting the exercises. | Physiotherapist | 24 in 8W |
| Jeyakumar, 2017 | EX+MT  (maitland) 20  EX+MT (mobilization with movement) 20  EX 20 | Group A received Mulligan’s MWM including medial, lateral and rotation MWM without a belt with a frequency of one glide per second for 30 seconds. Group B received Maitland’s MWM including medial, lateral and posterior glide with the frequency of one glide per second for 30 seconds. | Group C received conventional physiotherapy which includes hot packs, isometric quadriceps exercises, and straight leg raise. | Physiotherapists | NR  NR |
| ko, 2009 | EX 17  EX+MT 18 | First, mobilization by pulling the knee joint was done twice for 30 s, at the end of the extension and flexion creak. Then, mobilization by extending the knee joint (previous gliding) was done twice for 30 s, in the range of motion and at the end of the extension creak. Next, mobilization by flexing the knee joint (posterior gliding) was done twice for 30 s, in the range of motion and at the end of the flexion range. Finally, mobilization by the gliding of the patella (distal gliding) was done twice for 30 s, in the range of motion and at the end of the flexion range. The stage of mobilization for manual therapy was adjusted according to the state of the joint and the patient’s response, and the intensity was controlled by sensitively judging the range of motion, the quality of motion, and the joint end-feel. | Resistive exercise was performed twice by the  subjects in the RT program with a 20-s break given  after each exercise. First, while the knee joint was  extended, the subjects maintained static tension in  the quadriceps femoris muscle for 6 s and then took a 10-s break; this process was repeated 10 times.  Next, they stood with the knee joint extended to the end of the range and repeated a knee-extension  exercise using an elastic band (yellow), 12 times for 1.5 min. Afterwards, they sat and repeated the leg press, using an elastic band (yellow) 12 times for 1.5 min. Then, they undertook a 1-s concentric contraction and a 2-s eccentric contraction with  both legs, for 1.5 min; if they were able to, they  were asked to repeat the same exercise with one leg. Finally, they performed step-ups 12 times, for 1.5 min; if they were able to, they were asked to  increase the steps height. The resistance of the  elastic band differs with the length of extension,  and a 3.5lb (1.5876-kg) resistance is generated by  extending the yellow elastic band by 67%. The  permissible amount of exercise was defined as  exercise which could be performed for more than 30min without causing pain in the joint. | NR | 24 in 8W  40min |
| Kulkarni, 2017 | EX 15  EX+MT 15 | The experimental group received conventional treatment along with the Mulligan mobilization technique. This group received all conventional treatment (as explained above) along with the Mulligan mobilization technique. | The control group received conventional treatment (TENS and exercise program). Conventional 2-pole TENS with a frequency of 100 Hz was administered for 15-20 minutes for 3 days starting on the first day. Patients were treated in a supine position with rotation under the affected knee. | Physiotherapists | 3 in 3D  NR |
| Lalit, 2012 | EX+MT (maitland) 30  EX+MT (mobilization with movement) 30  EX 30 | Maitland: Anterior and posterior tibiofemoral and superior-inferior and mediolateral patellofemoral glides were given. Degree of mobilization: Grade 2 and 3.  Muligan: The therapist flexed the knee just short of the painful limitation. Now the therapist grasped the lower leg and internally rotated the tibia on the femur. The patient was then asked to flex further and overpressure was applied. Dosage: 3 sets of 3 repetitions each. | Multiple Angle Isometric Exercises  2. Terminal Arch Knee Extension  3. Mini Squat  4. Partial Lunges  5. Unilateral Balances  6. Cross-Leg Swings  Repetitions: 10 repetitions per session  3 sessions per day. | Physiotherapist | 120 in 8W  NR |
| Lalnunpuii, 2017 | EX+MT (mobilization with movement) 15  EX+ MT (maitland) 15  EX 15 | Mulligan mobilization consisted of:  Medial glide mobilization with movement, lateral glide mobilization with movement, rotational mobilization with movement, and dorsal glide with active knee flexion depending on the subject's condition. The glide force was sustained while the patient performed 3 sets of 10 repetitions.  Maitland's technique consisted of Grade II & III glides performed on the following joints/direction/maneuver according to the subject's condition - Anteroposterior tibiofemoral glide, tibiofemoral glide, posteroanterior glide, patellofemoral caudal-cephaloid glide. 2 or 3 oscillations per second for 1 to 2 minutes was performed. | The supervised exercise program consisted of  isometric strengthening of quadriceps, hamstrings and  VMO with a 15-second hold, 4 sets of 10  repetitions, flexibility exercises such as quadriceps  stretching, hamstring stretching for 30 seconds and repeated  3 times, knee range of motion exercises, such as mid  flexion to full extension, mid flexion to full flexion two 30  second fights with a 3-second hold at the final interval were  performed. These exercises were performed by all  subjects under the supervision of the therapist. | Physiotherapists | 12 in 4W  NR |
| Mehwish, 2023 | TM+EX (hip and knee) 22  EX (hip and knee) 22  EX (knee) 22 | Hip mobilizations (anterior posterior glide,  posterior anterior glide, caudal glide, posterior anterior glide with abduction, flexion and lateral rotation in 3 sets; 120 oscillations per minute). Grades were assigned from 1-4 depending on the tolerance level with duration of 30-second rest in between14 along with strengthening exercises of hip (hip abduction in side lying, hip extension in prone position, sideway walk, hip abduction in a stand up position and hip hitting with a frequency of 2-3 sets; 10-15 repetitions in each set). | Evidence-based knee exercises (knee strengthening exercises, including knee extension static quads sets, terminal knee extension while standing, sitting leg press, step-ups; stretching exercises  including calf, hamstring, and quadriceps stretch; ROM exercises, including flexing knee mid-range to full and flexing knee mid-range to full).11 All exercises were performed in 10 repetitions, 3 sets with a remainder of 5 seconds in between. | NR | 8 in 4W  30-40min |
| Mishel, 2013 | EX+shock waves 15  EX+MT (mobilization with movement) 15  EX 15 | According to the work of Mulligan's and  Abdel-razek, MWM was conducted as follows.  a- Medial glide Mobilization with movement  The patient lied prone and to apply medial glide the therapist stood on the contralateral side, placed a belt around his waist and the patient's lower leg so that the proximal edge was at the tibial joint margin. The therapist stabilized the thigh above the knee with one hand and supported the lower leg with the other. The therapist glided the knee medially with the belt and asked the patient to flex his knee.  b- Lateral glide Mobilization with movement  The patient lied prone and to apply lateral glide the therapist stood on the same side, placed a belt around his waist and the patient's lower leg so that the proximal edge was at the tibial joint margin. The therapist stabilized the thigh above the knee with one hand and supported the lower leg with the other. The therapist glided the knee laterally with the belt and asked the patient to flex his knee.  c-Rotation mobilization with movement  A patient can do his own rotation MWMS with the painful leg on a chair. He placed his hands proximally around the lower leg and rotated his tibia medially. After sustaining this rotation he bent forward to flex his knee (weight-bearing) provided there was no pain. The hands on the fibular side carried it forward at the same time  d- MWM. Dorsal glide with active knee  flexion  The patient lied supine and the therapist stood beside the knee to be treated. With his fingers interlaced placed his hands over the flexed knee so that the heel of one hand lied over the tibial plateau, and the heel of the other was over the lower end of the femur. He glided the upper end of the tibia posteriorly when he was trying to proximate the heels of his hands. | The exercise program consisted of stretching the hamstring and calf muscles and straight leg raise exercises, three sets of 10 repetitions and isometric strengthening of the quadriceps muscle in the form of 3 submaximal isometric contractions of increasing intensity. | Physiotherapists | 12 in 4W  NR |
| Nafees, 2023 | EX+MT 24  EX 24 | Intervention group 'A' received dynamic soft tissue mobilization technique (DSTM).  1. Assessing the tight muscle group: The patient was prone lying. The physiotherapist applied a few deep strokes longitudinally on the hamstring muscle to assess the exact tightened part of the hamstring muscle and to limit the treatment to that specific area.  2. Dynamic intervention: The patient was in a supine lying with 90 ̊ flexion of the knee and hip joint. The physiotherapist applied the deep longitudinal strokes from the distal towards the proximal direction along with the tightened area of the hamstring muscle group while passively stretching the leg with the other hand in the direction of the hamstring lengthened position. The physiotherapist applied five longitudinal strokes and 20 s shaking at the end of this technique.  3. Then progressed to the next dynamic technique in the above-mentioned sequence but the physiotherapist asked the patient to do leg extension actively to achieve reciprocal inhibition of the hamstring muscle.  4. Then in the next technique, the patient applied a force to the physiotherapist’s hand to engage the hamstring eccentrically as the muscle was lengthened to its end range. In that position, the physiotherapist was applying 5 deep longitudinal strokes from distal towards the proximal direction on the tight hamstring muscle. | Intervention group 'B' received PNF stretching while cryotherapy and isometric strengthening exercises were applied to both groups.  Cryo therapy: Both groups received cold pack on anterior knee joint for 10 min.  Isometric Quadriceps Strengthening Exercises: These were given to both groups as the gold standard. The patient was lying supine. The physiotherapist placed a knee roll beneath the knee and instructed the patient to press the roll by using the back of the knee joint. The press was hold for 10 s following relaxed. Two sets of 10 repetitions  were performed.  Isometric Hip Adductor Strengthening Exercises:  These were also given to both groups. The patient was lying supine with both knees flexed. The physiotherapist placed a knee roll between the knee and instructed the patient to press the roll by joining both knees. The press was hold for 10 s following relaxed. About 2 sets of 10 repetitions were performed. | Physiotherapist | 12 in 4W  30min |
| Nigam, 2021 | EX+MT 20  EX 20 | A group receiving mobilization with movement  in addition to exercise and moist heat (Experimental group). With the patient lying in the supine position, the therapist applied a painless application of sustained manual gliding force to the proximal tibia near the knee joint (with counterforce on the femur) either in a lateral, medial, rotational, anterior or posterior direction. | One group received exercise alone and moist heat (control group). The exercises included pelvic bridge, resisted knee flexion and extension, mini squats and heel raises. | Physiotherapist | 6 in 2W  45min |
| Reza, 2021 | EX 16  EX+MT 16 | Group A  • Manual therapy including myofascial mobilization and manipulation techniques  • Myofascial mobilization technique  • Myofascial manipulation technique. | Supervised exercise protocol containing strengthening and stretching exercises  • Strengthening exercises include a series/group of exercises such as  • Static quadruple set in knee extension  • Standing terminal knee extension  • Seated leg press  • Partial squats (with weight reduction with arm support as needed) and  • Steps  • Stretching exercises include stretching different muscle groups in different positions, such as  • Standing calf stretch  • Hamstring stretch in supine position  • Quadriceps femoris stretch in prone position | NR | 3 in 2W  NR |
| Rojas-Vidal, 2018 | EX 10  EX+MT 10 | The conventional treatment proposed by MINSAL included tibiofemoral distraction, with a total session time of 40 minutes. Briefly, a warm, moist compress was applied for the first 10 minutes to prepare the tissues, followed by the tibiofemoral distraction technique for 5 minutes, then 10 minutes of ergometric cycling and 10 minutes of lower limb muscle strengthening exercises (LEII), concluding with 5 minutes of tibiofemoral distraction application. | The conventional treatment proposed by MINSAL includes applying a warm, moist compress for 10 minutes, using a stationary bicycle for 10 minutes, and concluding with LS muscle strengthening for 10 minutes. The treatment was performed 3 times a week for a total of 10 sessions. | Physiotherapist | 10 in 4W  40min |
| Ughreja, 2017 | EX+MT (mobilization with movement) 15  EX 15 | Subjects in group A received an intervention that consisted of MWM medial glide or MWM medial rotation (whichever is most indicated for pain relief for a given patient). | It consisted of shortwave diathermy for 10 minutes and conventional exercises. In all strengthening exercises, each contraction was held for ten seconds followed by a 3-second rest and repeated ten times. | Physiotherapist | 7 in 1W  NR |
| Zeya, 2017 | EX+MT 30  EX 30 | Joint Mobilization  Soft Tissue Manipulation  One experimental group received manual therapy and clinical exercises under the supervision of a physical therapist. | Strengthening  Stretching  Aerobic  The other experimental group was given a detailed exercise program to be performed at home. | NR | NR  NR |

**Legend: MT:** Manual Therapy; **EX:** Exercise; **B:** Booster session; **TENS:** Transcutaneous electrical nerve stimulation; **PNF:** Proprioceptive neuromuscular facilitation; **NR:** Not Reported; **D:** Day; **W:** Week; **M:** Month.

**Appendix S4:** TUG - A - medium-term and B - long-term

**Appendix S5:** 40m Fast Paced Walk Test - A - medium-term and B - long-term

**Appendix S6:** 6MWT - A - short-term and B - medium-term

**Appendix S7:** Stair climb test - Short-term.

**Appendix S8:** Proprioception - Short-term.

**Appendix S9:** 30s Chair Sit-to-Stand - Long-term.

**Appendix S10:** GRADEpro

| **Certainty assessment** | | | | | | | **№ of patients** | | **Effect** | | **Certainty** | **Importance** |
| --- | --- | --- | --- | --- | --- | --- | --- | --- | --- | --- | --- | --- |
| **№ of studies** | **Study design** | **Risk of bias** | **Inconsistency** | **Indirectness** | **Imprecision** | **Other considerations** | **[MT+EX]** | **[EX]** | **Relative  (95% CI)** | **Absolute  (95% CI)** |  |  |
| **IMMEDIATE PAIN (follow-up: mean 1 days; assessed with: END; Scale from: 0 to 10)** | | | | | | | | | | | | |
| 3 | randomised trials | very serious^a^ | not serious | not serious | serious^b,c^ | publication bias strongly suspected^d^ | 113 | 80 | - | MD **0.49 pontos fewer**  (0.92 fewer to 0.06 fewer) | ⨁◯◯◯  Very low^a,b,c,d^ | CRÍTICO |
| **SHORT PAIN (follow-up: mean 4 weeks; assessed with: END; Scale from: 0 to 10)** | | | | | | | | | | | | |
| 15 | randomised trials | serious^e^ | serious^f^ | not serious | not serious | none | 355 | 288 | - | MD **1.28 pontos fewer**  (1.71 fewer to 0.84 fewer) | ⨁⨁◯◯  Low^e,f^ | CRÍTICO |
| **MEDIUM PAIN (follow-up: mean 6 months; assessed with: END; Scale from: 0 to 10)** | | | | | | | | | | | | |
| 8 | randomised trials | not serious | serious^g^ | not serious | not serious | publication bias strongly suspected^d^ | 318 | 318 | - | MD **0.98 PONTOS fewer**  (1.86 fewer to 0.1 fewer) | ⨁⨁◯◯  Low^d,g^ | CRÍTICO |
| **LONG PAIN (follow-up: mean 2 years; assessed with: END; Scale from: 0 to 10)** | | | | | | | | | | | | |
| 4 | randomised trials | not serious | not serious | not serious | not serious | publication bias strongly suspected^d^ | 286 | 294 | - | MD **0.15 PONTOS more**  (0.01 more to 0.29 more) | ⨁⨁⨁◯  Moderate^d^ | CRÍTICO |
| **SHORT WOMAC (follow-up: mean 4 weeks; assessed with: QUESTIONÁRIO; Scale from: 0 to 1000)** | | | | | | | | | | | | |
| 8 | randomised trials | serious^e^ | very serious^g^ | not serious | serious^b^ | publication bias strongly suspected^d^ | 200 | 185 | - | SMD **0.83 SD fewer**  (1.38 fewer to 0.29 fewer) | ⨁◯◯◯  Very low^b,d,e,g^ | CRÍTICO |
| **MEDIUM WOMAC (follow-up: mean 6 months; assessed with: QUESTIONARIO; Scale from: 0 to 1000)** | | | | | | | | | | | | |
| 7 | randomised trials | not serious | very serious^g^ | not serious | not serious | publication bias strongly suspected^d^ | 255 | 256 | - | SMD **0.56 SD fewer**  (1.04 fewer to 0.07 fewer) | ⨁◯◯◯  Very low^d,g^ | CRÍTICO |
| **LONG WOMAC (follow-up: mean 2 years; assessed with: QUESTIONARIO; Scale from: 0 to 1000)** | | | | | | | | | | | | |
| 4 | randomised trials | not serious | serious^f^ | not serious | serious^h^ | publication bias strongly suspected^d^ | 285 | 291 | - | SMD **0.09 SD fewer**  (0.34 fewer to 0.17 more) | ⨁◯◯◯  Very low^d,f,h^ | CRÍTICO |
| **MEDIUM TUG (follow-up: mean 6 months; assessed with: SEGUNDOS)** | | | | | | | | | | | | |
| 4 | randomised trials | not serious | very serious^g^ | not serious | serious^i^ | publication bias strongly suspected^d^ | 229 | 230 | - | MD **1.73 SECONDS fewer**  (3.73 fewer to 0.28 more) | ⨁◯◯◯  Very low^d,g,i^ | CRÍTICO |
| **LONG TUG (follow-up: mean 2 years; assessed with: SEGUNDOS)** | | | | | | | | | | | | |
| 4 | randomised trials | not serious | serious^f^ | not serious | serious^i^ | publication bias strongly suspected^d^ | 287 | 294 | - | MD **0.27 SECONDS more**  (0.29 fewer to 0.83 more) | ⨁◯◯◯  Very low^d,f,i^ | CRÍTICO |
| **MEDIUM 40M FAST PACED WALK TEST (follow-up: mean 6 months; assessed with: SEGUNDOS)** | | | | | | | | | | | | |
| 2 | randomised trials | serious^j^ | very serious^g^ | not serious | not serious | publication bias strongly suspected^d^ | 200 | 201 | - | MD **0.66 SECONDS fewer**  (1.81 fewer to 0.48 more) | ⨁◯◯◯  Very low^d,g,j^ | CRÍTICO |
| **LONG 40M FAST PACED WALK TEST (follow-up: mean 2 years; assessed with: SEGUNDOS)** | | | | | | | | | | | | |
| 4 | randomised trials | not serious | very serious^g^ | not serious | not serious | publication bias strongly suspected^d^ | 286 | 294 | - | MD **0.96 SECONDS more**  (1.27 fewer to 3.19 more) | ⨁◯◯◯  Very low^d,g^ | CRÍTICO |
| **SHORT 6MWT (follow-up: mean 4 weeks; assessed with: METROS)** | | | | | | | | | | | | |
| 3 | randomised trials | not serious | serious^f^ | not serious | very serious^b,k^ | publication bias strongly suspected^d^ | 90 | 90 | - | MD **29.04 METERS more**  (11.89 fewer to 69.98 more) | ⨁◯◯◯  Very low^b,d,f,k^ | CRÍTICO |
| **MEDIUM 6MWT (follow-up: mean 6 months; assessed with: METROS)** | | | | | | | | | | | | |
| 2 | randomised trials | not serious | not serious | not serious | very serious^b,k^ | publication bias strongly suspected^d^ | 70 | 69 | - | MD **19.22 METERS more**  (31.4 fewer to 69.84 more) | ⨁◯◯◯  Very low^b,d,k^ | CRÍTICO |
| **SHORT STAIR CLIMB TEST (follow-up: mean 4 weeks; assessed with: SECONDS)** | | | | | | | | | | | | |
| 2 | randomised trials | not serious | very serious^g^ | not serious | serious^b^ | publication bias strongly suspected^d^ | 27 | 26 | - | SMD **1.22 SD fewer**  (3.52 fewer to 1.09 more) | ⨁◯◯◯  Very low^b,d,g^ | CRÍTICO |
| **SHORT PROPRIOCEPTATION (follow-up: mean 4 weeks; assessed with: GRAUS)** | | | | | | | | | | | | |
| 2 | randomised trials | very serious^l^ | serious^f^ | not serious | very serious^b,h^ | publication bias strongly suspected^d^ | 80 | 50 | - | SMD **0.28 SD fewer**  (0.64 fewer to 0.08 more) | ⨁◯◯◯  Very low^b,d,f,h,l^ | NÃO IMPORTANTE |
| **LONG 30S-CST (follow-up: mean 2 years; assessed with: N DE REPETIÇÕES )** | | | | | | | | | | | | |
| 4 | randomised trials | not serious | not serious | not serious | serious^m^ | publication bias strongly suspected^d^ | 286 | 294 | - | MD **0.1 STANDS fewer**  (0.23 fewer to 0.04 more) | ⨁⨁◯◯  Low^d,m^ | CRÍTICO |

**CI:** confidence interval; **MD:** mean difference; **SMD:** standardised mean difference

#### **Explanations**

a. >50% of participants in studies with a PEDro score <6
 b. Fewer than 400 participants in the comparison
 c. MD <10% of the scale
 d. Fewer than 10 studies
 e. >25% of participants in studies with a PEDro score <6
 f. I² greater than 50%
 g. I² greater than 75%
 h. SMD less than 0.5
 i. Value below the MIC (2.82 seconds)
 j. 101 participants in one study with a PEDro score <5
 k. Value below the MIC (61 meters)
 l. All participants in studies with a PEDro score <6
 m. Value below the MIC (2.5 repetitions)

**Appendix S11: Sensitivity Analysis**

| **Outcome** | **All studies** | **Removed studies without random allocation/allocation concealment** | **Co-interventions removed** | **Removed studies with mixed population** |
| --- | --- | --- | --- | --- |
|  | **MD/SMD [95% CI]; number of studies; I²** | **MD/SMD [95% CI]; number of studies; I²** | **MD/SMD [95% CI]; number of studies; I²** | **MD/SMD [95% CI]; number of studies; I²** |
| Pain (short-term) | -1.28 [-1.71, -0.84]; studies=15; I²=68% | -1.30 [-1.74, -0.87]; studies=14; I²=70% | -1.05 [-1.41, -0.69]; studies=9; I²=25% | NA |
| Pain (medium-term) | -0.98 [-1.86, -0.10]; studies=8; I²=96% | -0.95 [-2.07, 0.17]; studies=6; I²=97% | NA | NA |
| Pain (long-term) | 0.15 [0.01, 0.29]; studies=4; I²=0% | NA | NA | 0.16 [0.01, 0.30]; studies=2; I²=0% |
| Function (short-term) | -0.83 [-1.38, -0.29]; studies=8; I²=83% | -0.88 [-1.34, 0.43]; studies=5; I²=65% | -0.51 [-1.15, 0.12]; studies=5; I²=82% | NA |
| Function (medium-term) | -0.56 [-1.04, -0.07]; studies=7; I²=83% | NA | -0.58 [-1.31, 0.16]; studies=4; I²=90% | NA |
| Function (long-term) | -0.09 [-0.34, 0.17]; studies=4; I²=51% | NA | NA | -0.18 [-0.58, 0.22]; studies=2; I²=62% |

**Appendix S12: Funnel Plot**

**Appendix S13: Definition of manual therapy techniques**

According to the International Federation of Manual and Musculoskeletal Physical Therapists (IFOMPT), manual therapy (MT) comprises skilled hand movements intended to optimise any or all of the following effects: improve tissue extensibility; increase range of motion; mobilise or manipulate soft tissues and joints; induce relaxation; change muscle function; stabilise the joint complex; modulate pain; reduce soft tissue swelling, inflammation or movement restriction (IFOMPT Standards Document, 2016). Accordingly, MT encompasses a broad range of interventions, including joint mobilization, joint manipulation, soft tissue techniques (manual or instrument-assisted), massage, and acupressure, selected according to the patient's clinical presentation and treatment goals (IFOMPT Standards Document, 2016).
